# Supplementary material for: Coupling of palaeontological and neontological reef coral data improves forecasts of biodiversity responses under global climatic change
Source: R Soc Open Sci. 2019 Apr 24;6(4):182111. doi: 10.1098/rsos.182111 (PMC6502368; doi:10.1098/rsos.182111)
Supplement: Supplementary Material 2 [file rsos182111supp2.docx]

**Supplementary Material 2**

# Environmental data – Model description & data preparation

## HadCM3 – general circulation model

The atmospheric component of HadCM3 has a horizontal resolution of 2.5° x 3.75° and a vertical resolution of 19 levels, with a time step of 30 min. The horizontal resolution of the oceanic component has a higher resolution of 1.25° x 1.25°, with a vertical resolution of 20 levels to a depth of 5500 m. In this study, irradiance (atmospheric component) was bilinearly interpolated to 1.25° x 1.25° to match the resolution of the corresponding oceanic variables. HadCM3 is relatively low in resolution compared with more local-scale models, but it performs well, particularly in regards to mean climate state [1]. Although the resolution of environmental data might represent an aggregated value of the true value at occurrence localities, this is likely insignificant for parameters such as irradiance and sea surface temperature (SST), whereas salinity and bathymetry might vary quite considerably over small geographical distances [2]. However, previous work has shown salinity to be considerably less significant than both SST and irradiance at a global scale, with little contribution to model construction [3]. Nevertheless, at localised scales salinity and bathymetry are likely to have an impact upon the persistence of reef corals, and it should be borne in mind that it will not be captured within our ecological niche models (ENMs).

## Future climate scenarios

In this study, we used IPCC representative concentration pathways (RCP) 4.5 and 8.5 for our future climate scenario simulations [4-6]. RCP4.5 is a climate scenario simulation which stabilizes radiative forcing at 4.5 W m^−2^ (approximately equivalent to 650 ppm CO_2_) by the end of the century, without ever surpassing that value, whereas RCP8.5 has a radiative forcing of 8.5 W m^−2^ (approximately equivalent to 936 ppm CO_2_) by 2100 [5, 6]. The RCP4.5 scenario assumes that climate policies are put into practise to curb global greenhouse emissions, resulting in the stabilization of emissions [6]. Conversely, the RCP8.5 scenario assumes a global population of 12 billion by 2100, slow income growth, meek energy efficiency development, and little progress in curbing emissions [5].

## Digital elevation model

Bathymetric data were extracted from Getech’s present day digital elevation model (DEM), which provides gridded representations of both the Earth’s topography and bathymetry. This model has a resolution of 0.1° x 0.1°, which was resampled to 1.25° x 1.25° to be consistent with the resolution of the climate variables for ENM. To prevent the loss of shallow water areas surrounding small oceanic islands, we used a minimum-value resampling technique, assigning the minimum depth within the resampled area as the cell value. Preferably, we would utilise a Last Interglacial (LIG) DEM for constructing LIG ENMs and projections; however, accurate DEMs for this time are not available. Typically, the LIG eustatic sea level is thought to have been between 4 and 9 m higher than the present day [7-11]. We therefore carried out sensitivity studies to evaluate the effect of a heightened sea level at such large spatial scales, running models with sea level increased by 10 m (Figure SM1). We found no considerable difference in model output; as such, we used the modern DEM in both modern and LIG ENM constructs.


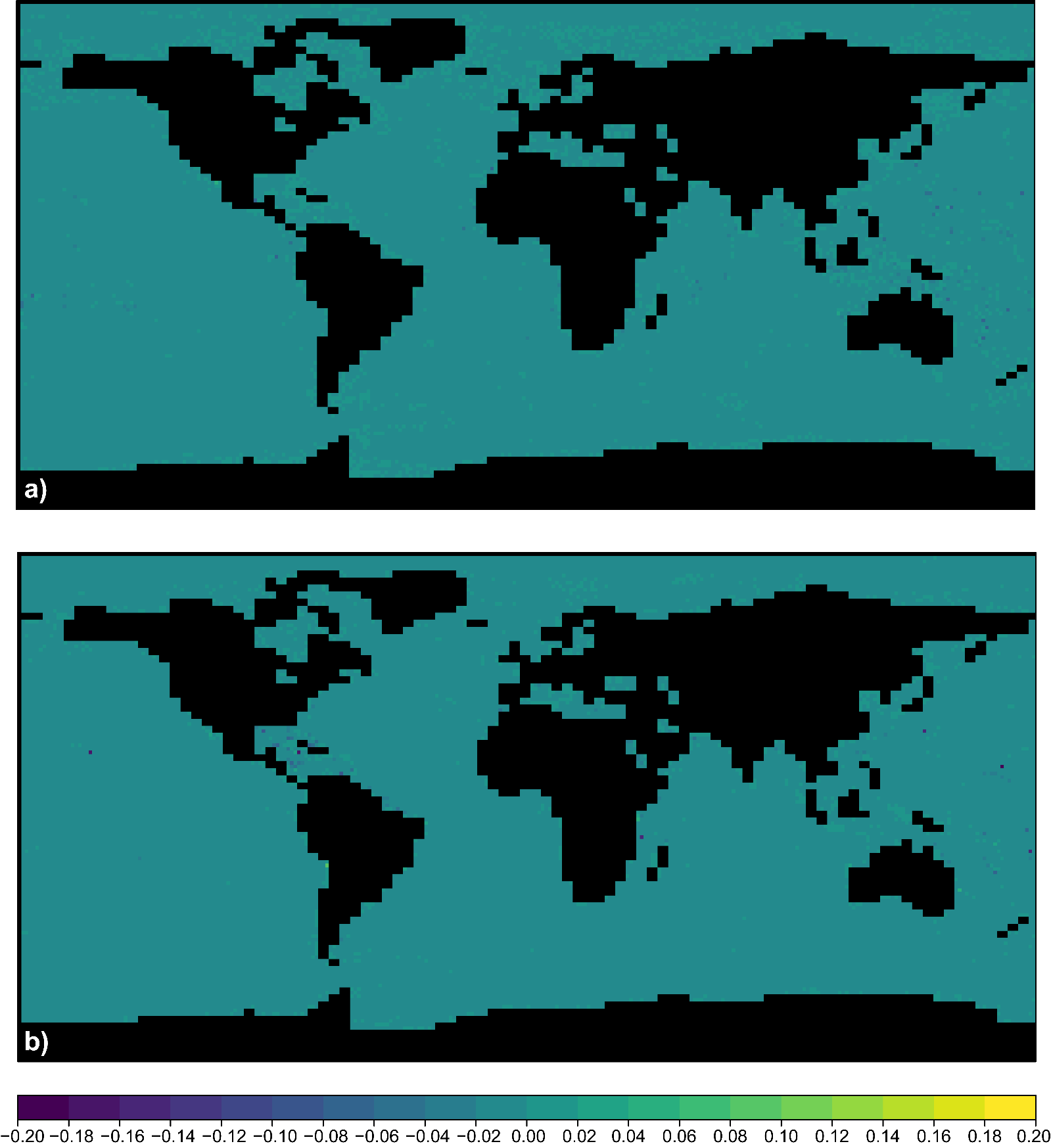


Figure SM 1. Difference maps of predicted habitability for the LIG. a) Modern trained ENM comparison of the LIG using the modern bathymetry and modern bathymetry + 10 m in LIG projections. b) LIG trained ENM comparison of the LIG using the modern bathymetry and modern bathymetry + 10 m for LIG training.

## Aragonite Saturation State

A number of variables considered to be of secondary significance in limiting the distribution of coral reefs, such as nutrient concentrations and aragonite saturation state, were not included in the generation of our ENMs [3]. This was largely due to these data being unavailable for the geological past or the future from general circulation model (GCM) outputs. However, as our analyses focus on reef corals, as opposed to actual reef frameworks, it is rational to conceive that aragonite saturation state is of lesser importance to the distribution of reef corals than it is coral reefs. Despite this, we carried out a sensitivity test comparing modern projections from a modern-trained ENM with and without aragonite saturation state included in the model (Figure SM2). As aragonite saturation state data were not available to us from GCM outputs, we used global gridded data from Jiang and Feely [12], calculated from a number of in situ measurements via a Matlab version of CO2SYS (see metadata within Jiang and Feely [12] for a full description on the data). We resampled and clipped this data in accordance to the environmental data used for ENM analyses. We found no considerable difference in global model projections for the modern (Figure SM2), with the exception of a few isolated regions (Timor Sea, South China Sea, and to a lesser extent, northern Andaman Sea [Myanmar], eastern Mediterranean Sea, and south-eastern Atlantic Ocean [Angola/Namibia]). Variable contribution analysis (Table SM1) confirms that aragonite saturation state is of secondary importance for global scale ENM. However, its contribution to ENM is not insignificant, with similar scores to June, July, August irradiance, and should therefore be considered within this context for the interpretation of ENM results.


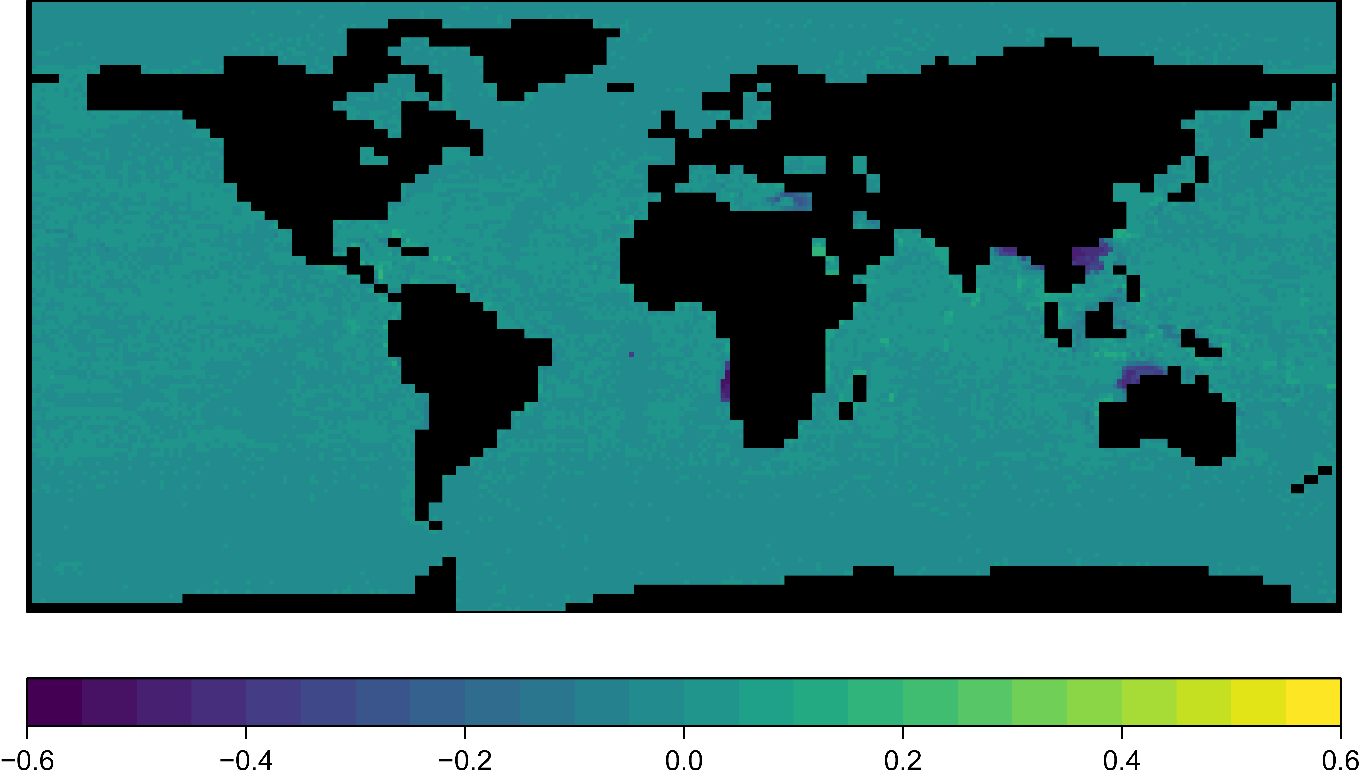


Figure SM 2. Difference map of predicted habitability for modern projections from modern-trained ENMs with and without aragonite saturation state variable included.

Table SM 1. Mean biomod2 variable importance test scores for modern-trained ENM with aragonite saturation state variable included. These results display 1 - cor (Pearson correlation coefficient score) between predictions based on calibrated models, and predictions after one environmental variable is randomised. Higher values indicate higher importance in model calibration, whist lower values indicate less importance.

| **ENVIRONMENTAL VARIABLE** | **MEAN VARIABLE CONTRIBUTION** |
| --- | --- |
| Aragonite saturation state | 0.14367 |
| Bathymetry | 0.81521 |
| December, January, February irradiance | 0.08713 |
| June, July, August irradiance | 0.13732 |
| Annual salinity | 0.05434 |
| Annual sea surface temperature | 0.43186 |

## Variable selection

Table SM 2. Ecologically relevant variables and their assigned short name and code used in this study.

| ENVIRONMENTAL VARIABLE | UNIT | SHORT NAME |
| --- | --- | --- |
| Annual irradiance | W/m^2^ | NETSWANN |
| April irradiance | W/m^2^ | NETSWAPR |
| August irradiance | W/m^2^ | NETSWAUG |
| December irradiance | W/m^2^ | NETSWDEC |
| Seasonal irradiance (December, January February) | W/m^2^ | NETSWDJF |
| February irradiance | W/m^2^ | NETSWFEB |
| January irradiance | W/m^2^ | NETSWJAN |
| Seasonal irradiance (June, July, August) | W/m^2^ | NETSWJJA |
| July irradiance | W/m^2^ | NETSWJUL |
| June irradiance | W/m^2^ | NETSWJUN |
| Seasonal irradiance (March, April, May) | W/m^2^ | NETSWMAM |
| March irradiance | W/m^2^ | NETSWMAR |
| May irradiance | W/m^2^ | NETSWMAY |
| November irradiance | W/m^2^ | NETSWNOV |
| October irradiance | W/m^2^ | NETSWOCT |
| September irradiance | W/m^2^ | NETSWSEP |
| Seasonal irradiance (September, October November) | W/m^2^ | NETSWSON |
| Annual salinity | mPSU | SALANN |
| Seasonal salinity (December, January, February) | mPSU | SALDJF |
| Seasonal salinity (June, July, August) | mPSU | SALJJA |
| Seasonal salinity (March, April, May) | mPSU | SALMAM |
| Seasonal salinity (September, October, November) | mPSU | SALSON |
| Annual sea surface temperature | °C | SSTANN |
| April sea surface temperature | °C | SSTAPR |
| August sea surface temperature | °C | SSTAUG |
| December sea surface temperature | °C | SSTDEC |
| Seasonal sea surface temperature (December, January, February) | °C | SSTDJF |
| February sea surface temperature | °C | SSTFEB |
| January sea surface temperature | °C | SSTJAN |
| Seasonal sea surface temperature (June, July, August) | °C | SSTJJA |
| July sea surface temperature | °C | SSTJUL |
| June sea surface temperature | °C | SSTJUN |
| Seasonal sea surface temperature (March, April, May) | °C | SSTMAM |
| March sea surface temperature | °C | SSTMAR |
| May sea surface temperature | °C | SSTMAY |
| November sea surface temperature | °C | SSTNOV |
| October sea surface temperature | °C | SSTOCT |
| September sea surface temperature | °C | SSTSEP |
| Seasonal sea surface temperature (September, October, November) | °C | SSTSON |


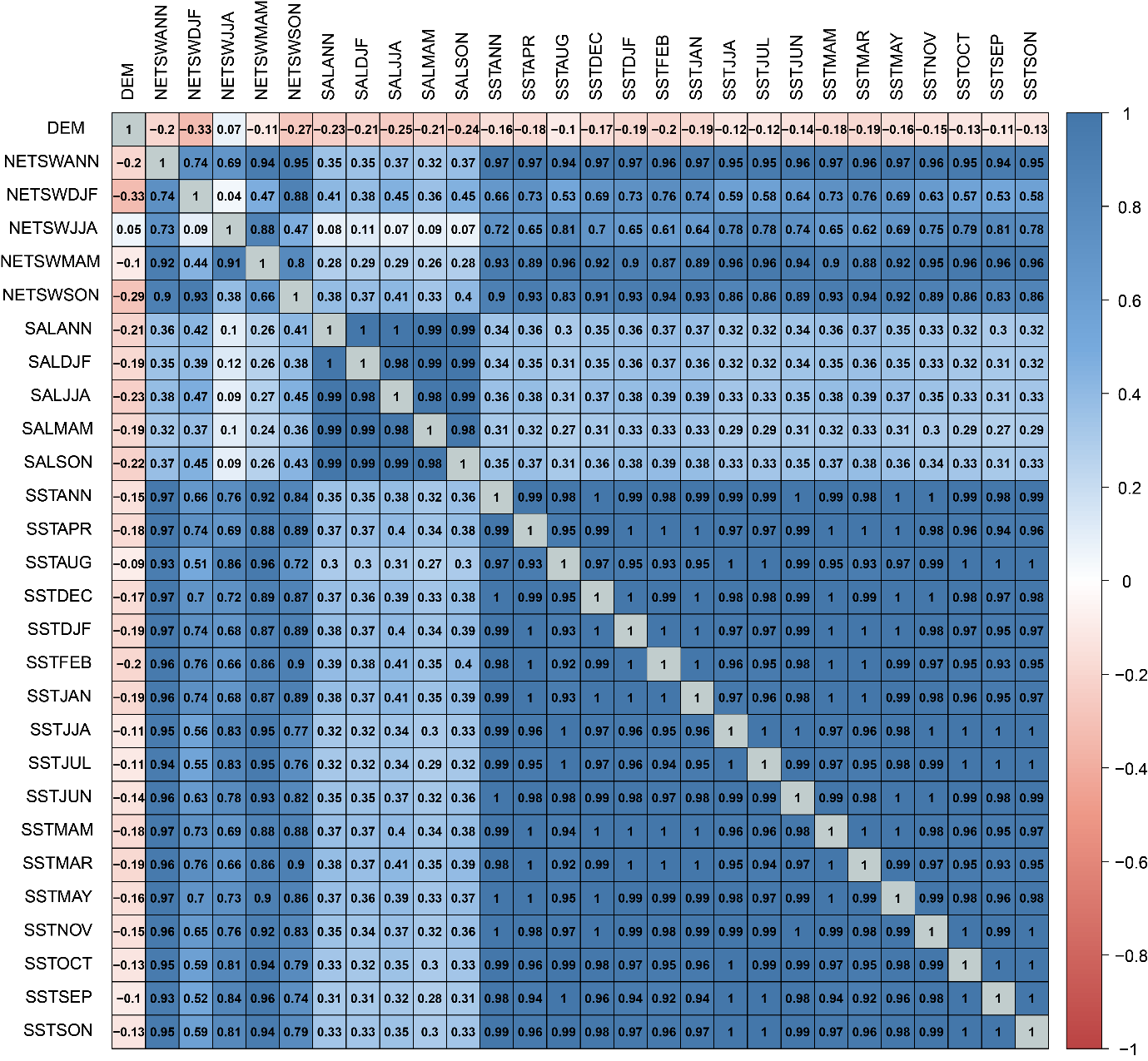


Figure SM 3. Correlation matrix of all modern and Last Interglacial environmental variables. The upper diagonal portion of the matrix shows the correlation coefficients of modern covariates. Whereas, the lower diagonal portion of the matrix shows the correlation coefficients of Last Interglacial covariates. Variable definitions are given in Table SM2. Scale bar represents Pearson’s correlation coefficient between each pair of variables (‘r’) in the grid.


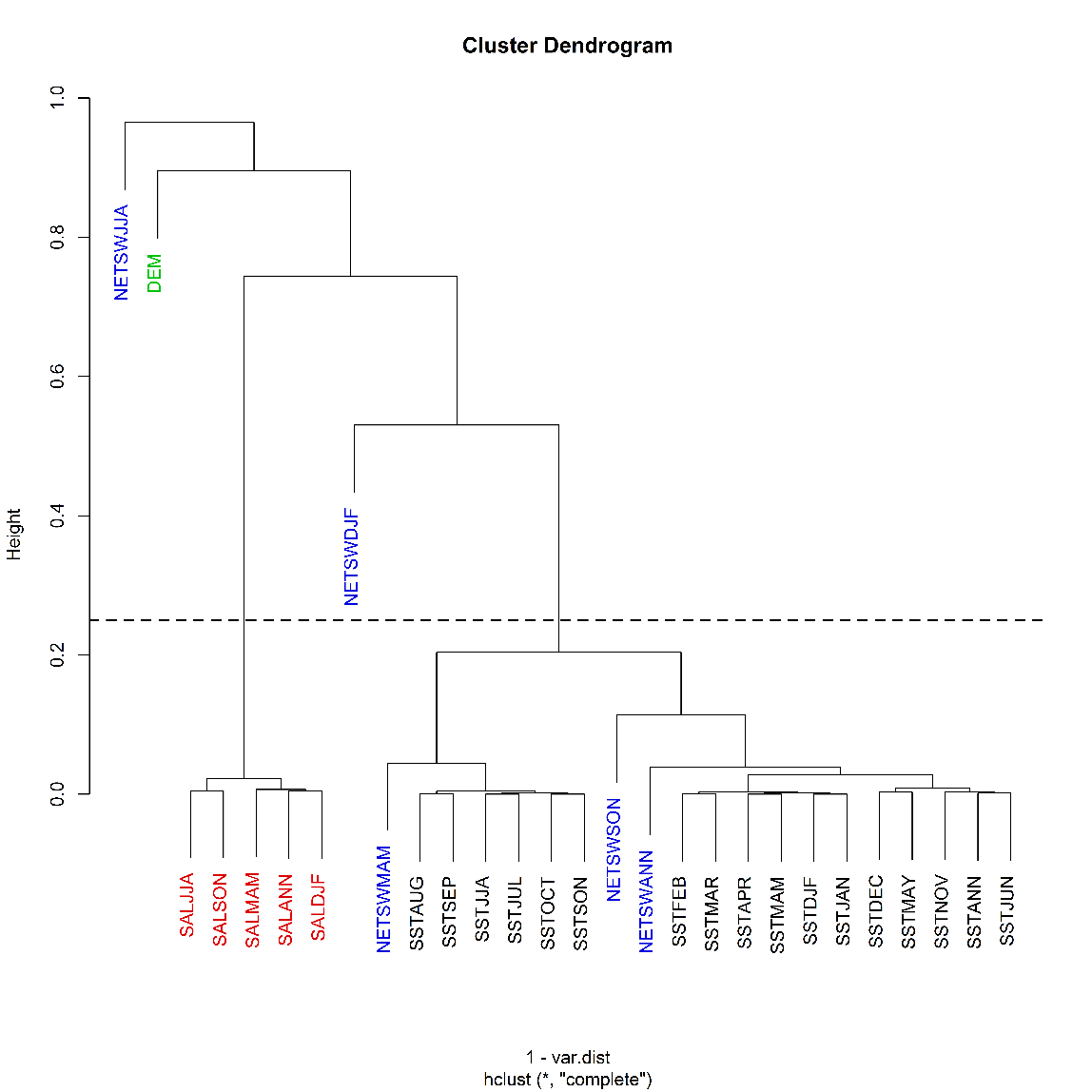


Figure SM 4. A cluster dendrogram illustrating the arrangement of clusters produced by hierarchical clustering for modern covariates. The height measure between clusters is calculated as H = 1 – C, where H = Height and C = correlation between compound clusters. Less height is equal to a higher correlation between clusters.


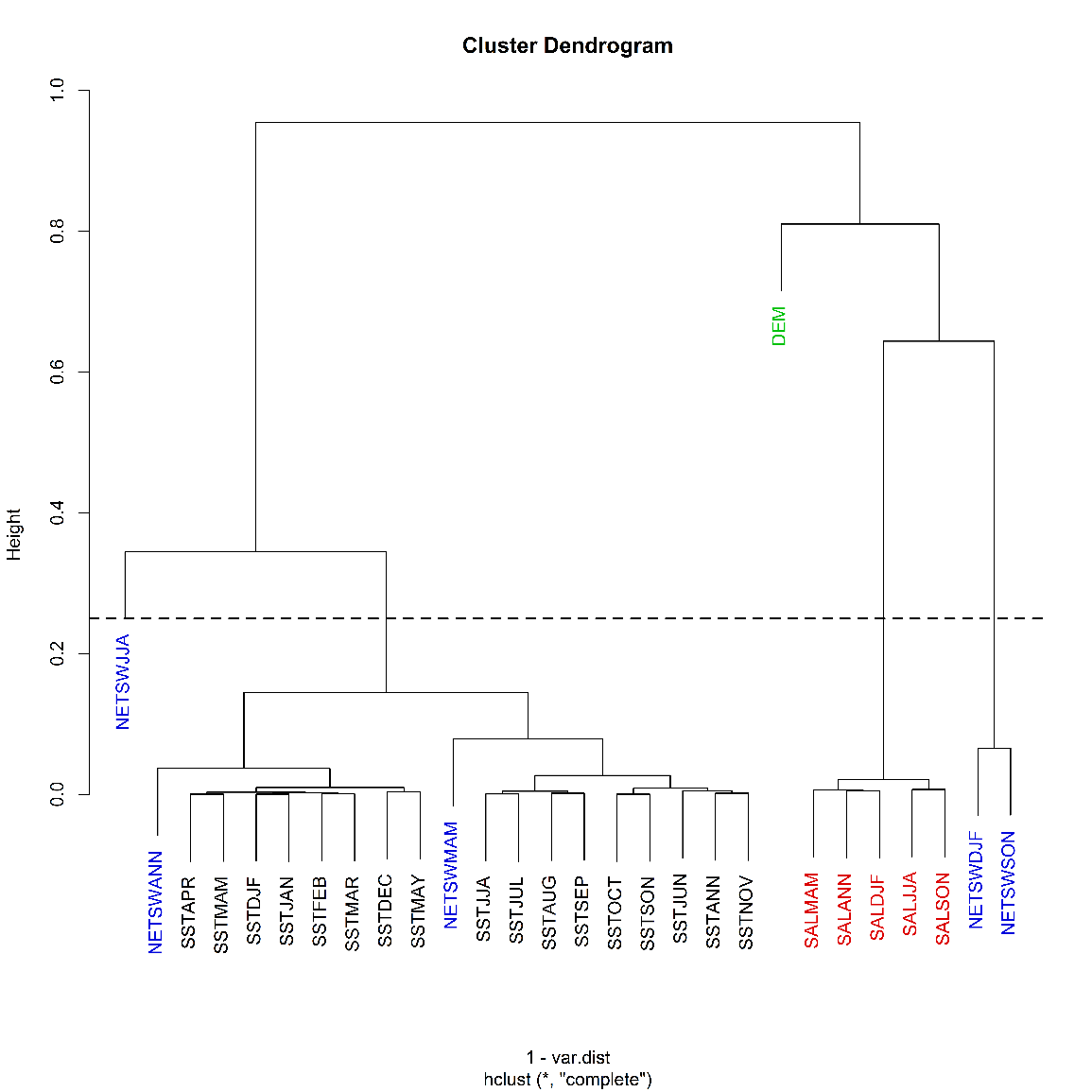


Figure SM 5. A cluster dendrogram illustrating the arrangement of clusters produced by hierarchical clustering for Last Interglacial covariates. The height measure between clusters is calculated as H = 1 – C, where H = Height and C = correlation between compound clusters. Less height is equal to a higher correlation between clusters.


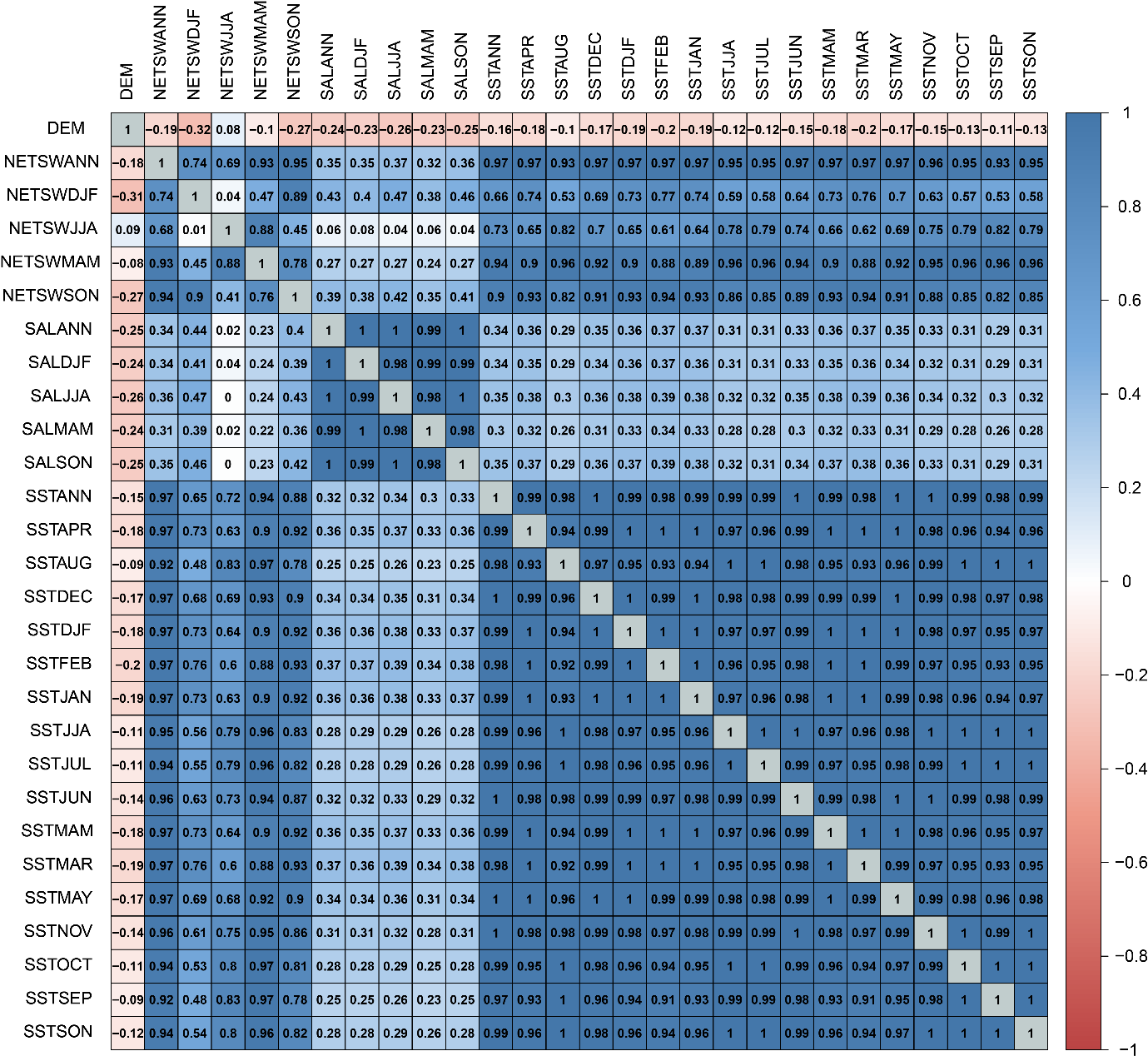


Figure SM 6. Correlation matrix of all RCP4.5 and RCP8.5 environmental variables. The upper diagonal portion of the matrix shows the correlation coefficients of RCP4.5 covariates. Whereas, the lower diagonal portion of the matrix shows the correlation coefficients of RCP8.5 covariates. Variable definitions are given in Table SM2. Scale bar represents Pearson’s correlation coefficient between each pair of variables (‘r’) in the grid.


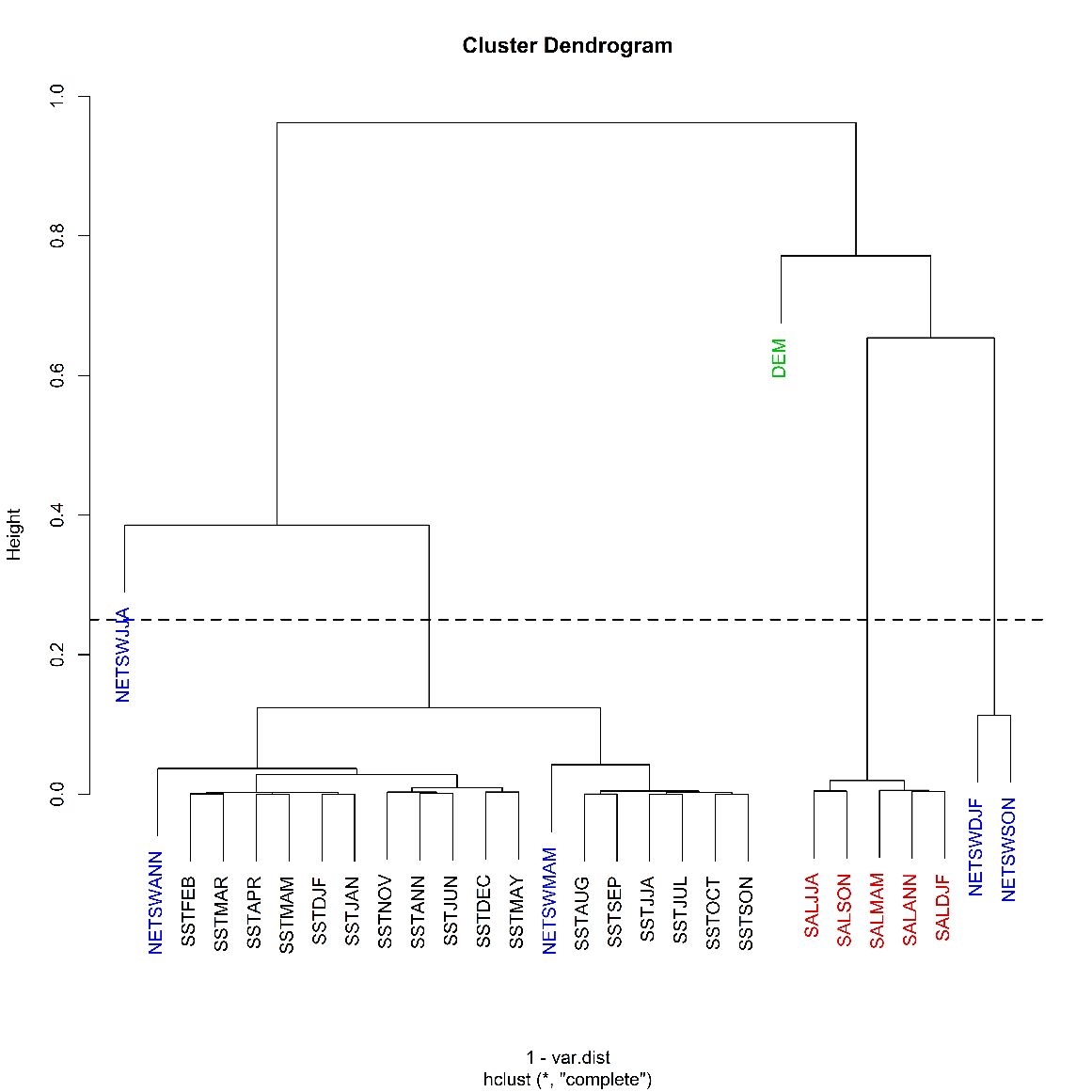


Figure SM 7. A cluster dendrogram illustrating the arrangement of clusters produced by hierarchical clustering for RCP4.5 covariates. The height measure between clusters is calculated as H = 1 – C, where H = Height and C = correlation between compound clusters. Less height is equal to a higher correlation between clusters.


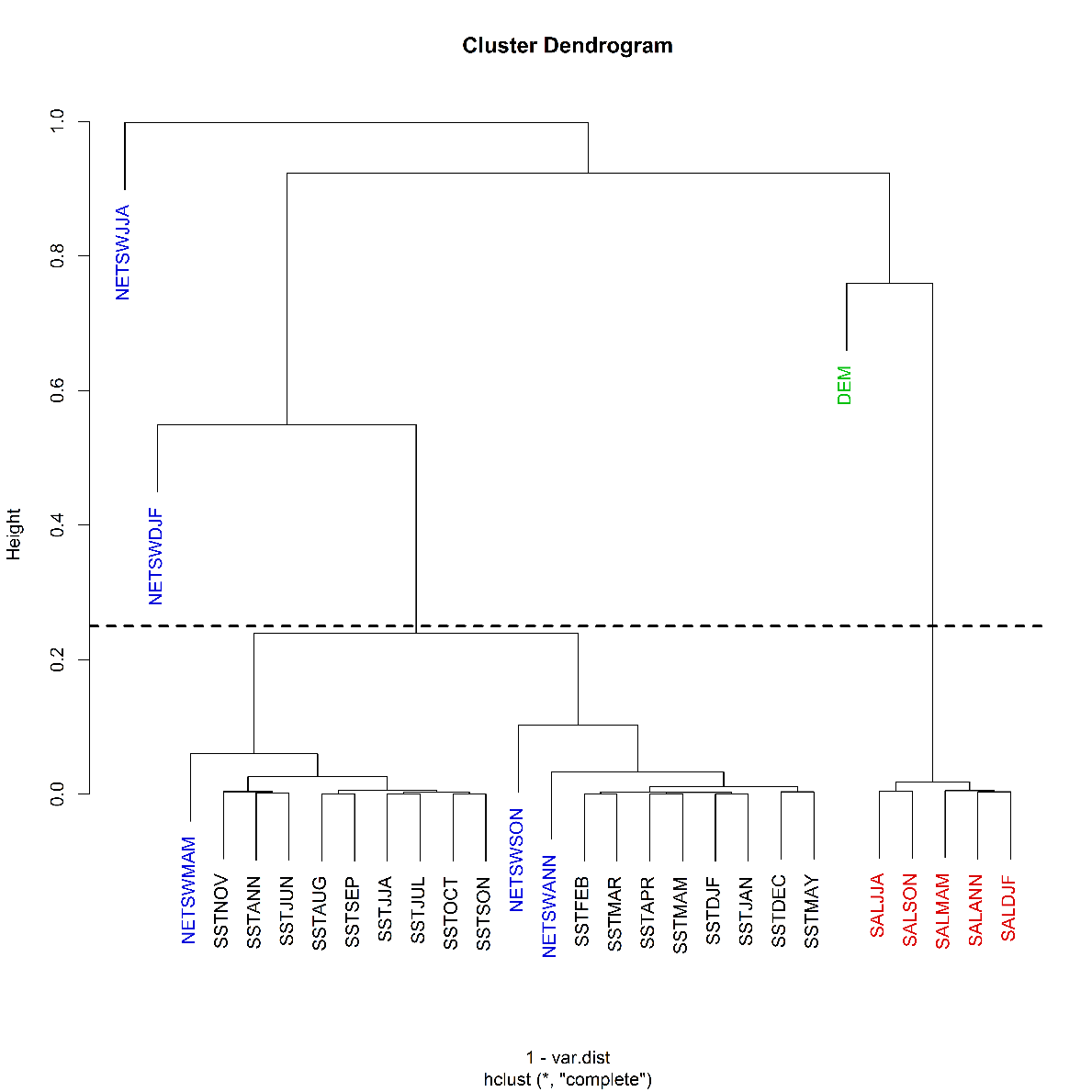


Figure SM 8. A cluster dendrogram illustrating the arrangement of clusters produced by hierarchical clustering for RCP8.5 covariates. The height measure between clusters is calculated as H = 1 – C, where H = Height and C = correlation between compound clusters. Less height is equal to a higher correlation between clusters.

# Niche analysis


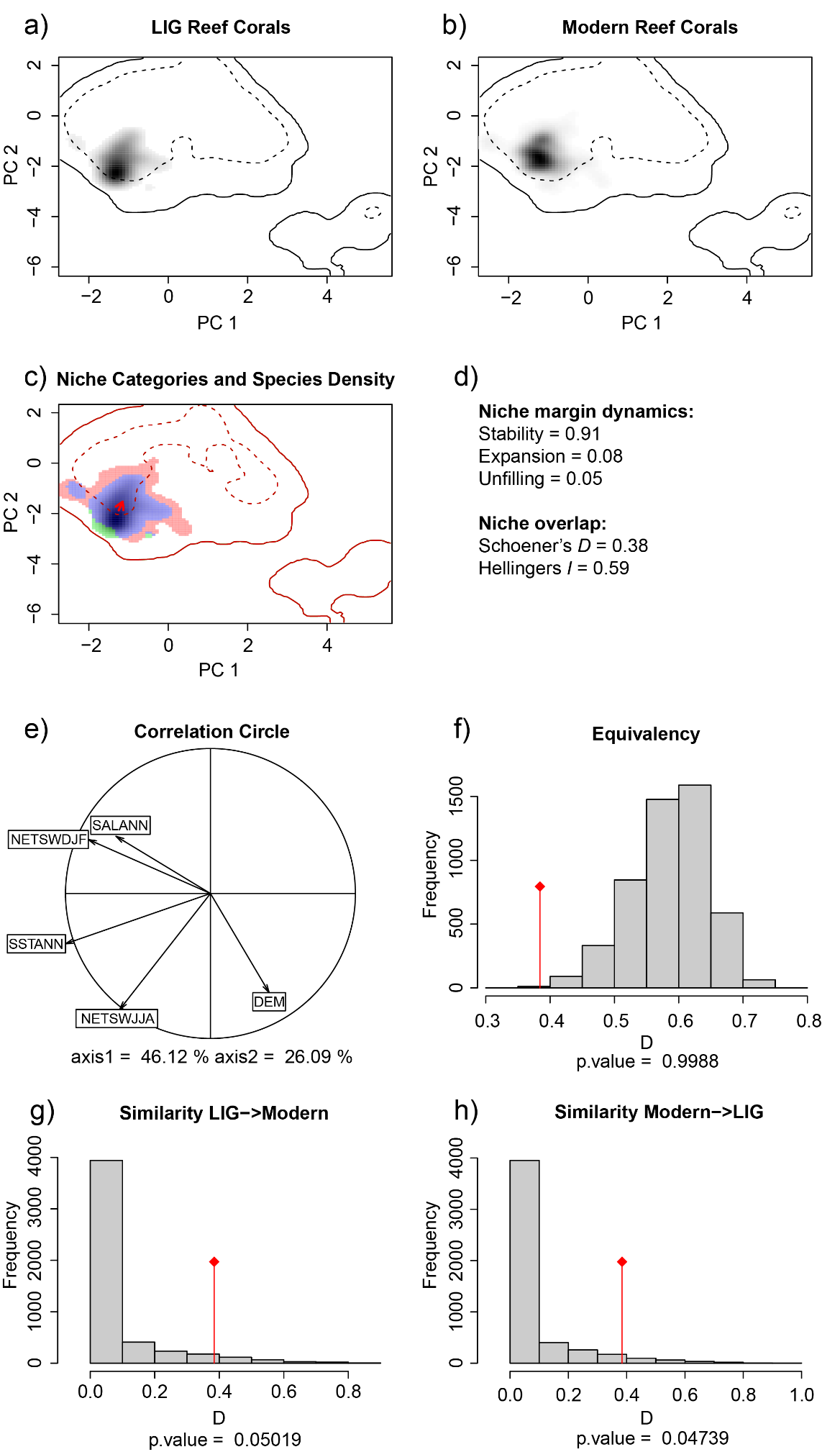


Figure SM 9. Niche overlap results for reef corals during the LIG and modern. (a-c) Comparison of the environmental space occupied by LIG and modern reef corals via the PCA-env. The figure shows considerable overlap (purple) in environmental space, minimal expansion (environmental space LIG reef corals did not occupy, but modern do [red]) and unfilling (environmental space LIG reef corals occupied but modern do not [green]). The solid contour line depicts 100% of environmental space available in both time slices, whilst the dashed line illustrates 50%. (c) The red arrow illustrates the directional change of the niche centre from LIG to modern. (d) Results of the niche margin dynamics and overlap tests. (e) Contribution of the environmental variables to the two axes of the PCA-env, as well as the percentage of inertia explained by the two axes. (f-h) The plots illustrate the results from both niche equivalency and similarity tests. The red diamond identifies the observed niche overlap metrics, whilst the grey bars indicates the randomly estimated niche equivalency/similarity.

# Ecological niche modelling

## Model validation and performance

To assess our model ensemble approach we determined the variation of calculated habitability between modelling methods. Accordingly, we extracted the values from model outputs for modern and LIG occurrence data to inspect the assigned raw habitability values, thus testing our approach rather than using an arbitrary threshold of 0.5. Habitability values for each occurrence record and model (Figure SM 10) validate our approach in the construction of binary maps due to the variance in assigned habitability values. BIOCLIM models appear to represent the most synchronous models between models trained on both the modern and LIG training dataset. However, the algorithm also assigns a large amount of the occurrences to low habitability scores. MaxEnt models show a general increase in occurrence records at higher habitability values, and appears mostly synchronous between models trained on both modern and LIG training datasets. RandomForest provides the most asynchronous results between models trained on both datasets. This suggests that within this study, RandomForest may be overfitting. This is particularly evident for those models trained on the LIG occurrence dataset, with a projection to the modern. These results, in combination with the AUC and TSS scores (Figure SM 11), suggest that MaxEnt performs better when compared to BIOCLIM and RandomForest across temporal boundaries, and potentially when analysing spatially biased training datasets.


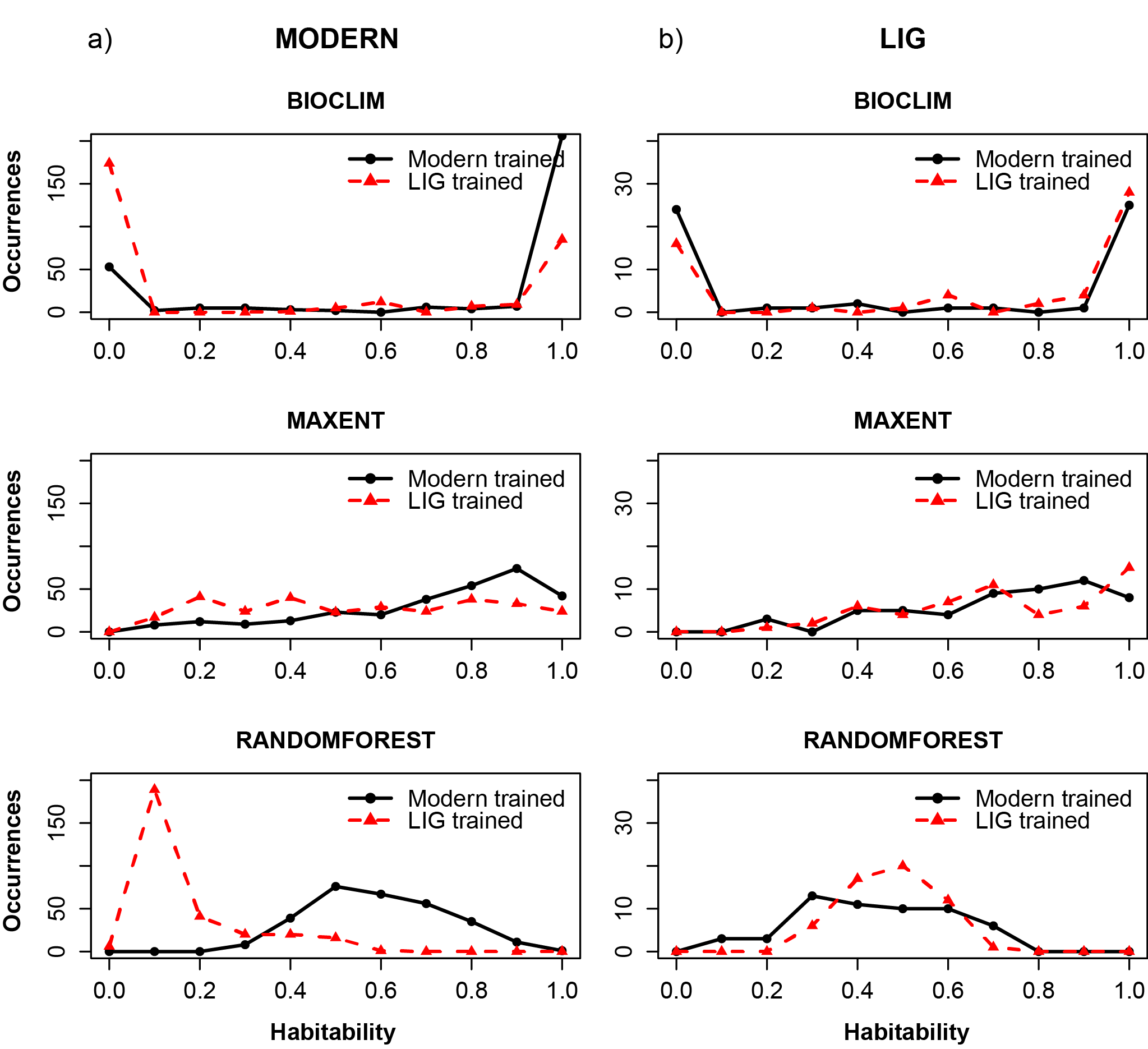


Figure SM 10. Binned habitability plots for each modelling algorithm’s habitability values associated with the known occurrences of modern and LIG reef corals. (a) Present day habitability values associated with modern occurrence records for models trained on the modern and LIG training dataset. (b) LIG habitability values associated with LIG occurrence records for models trained on the LIG and modern training dataset.


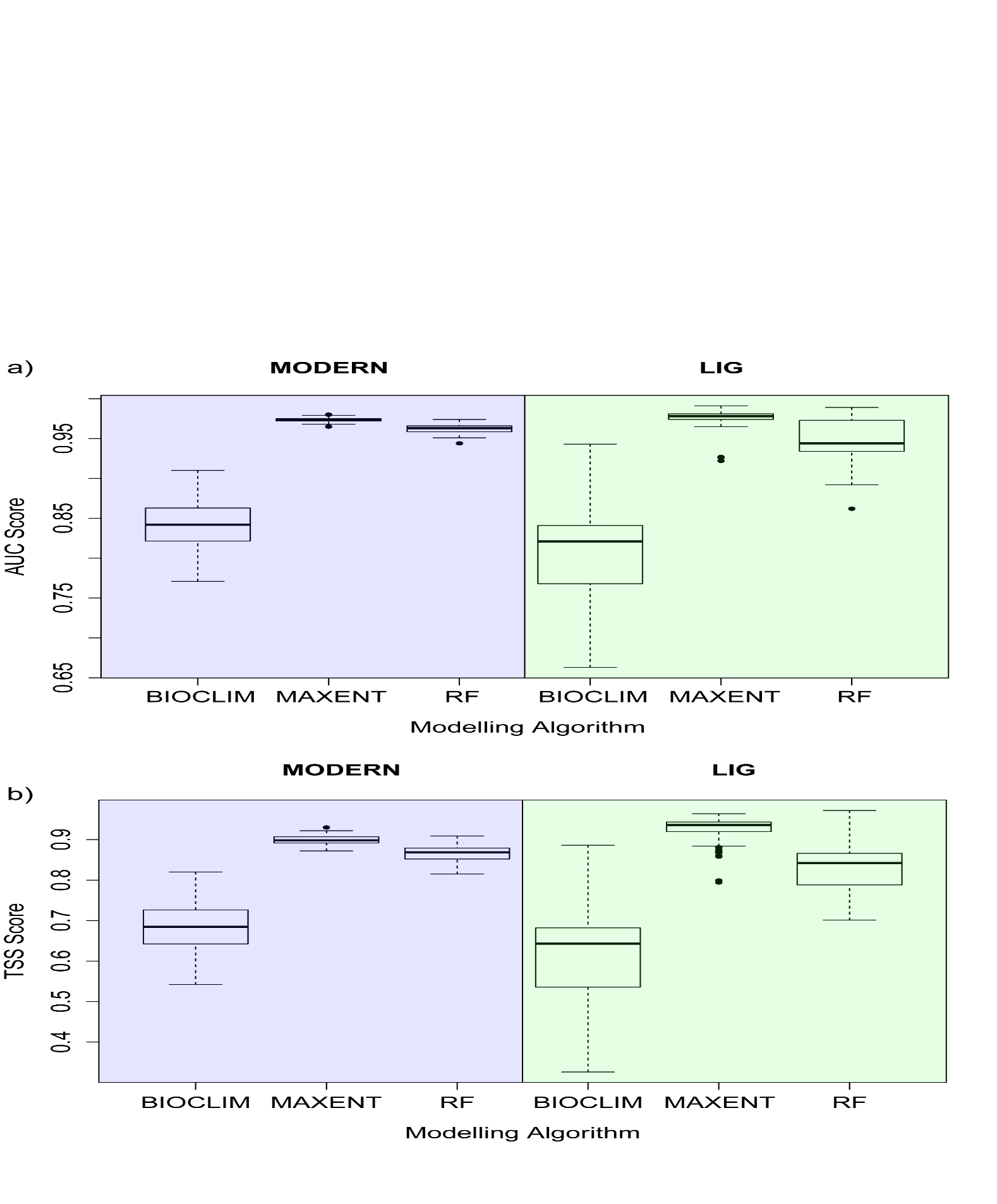


Figure SM 11. Boxplots illustrating model performance for each algorithm based on (a) AUC and (b) TSS scores for test data.


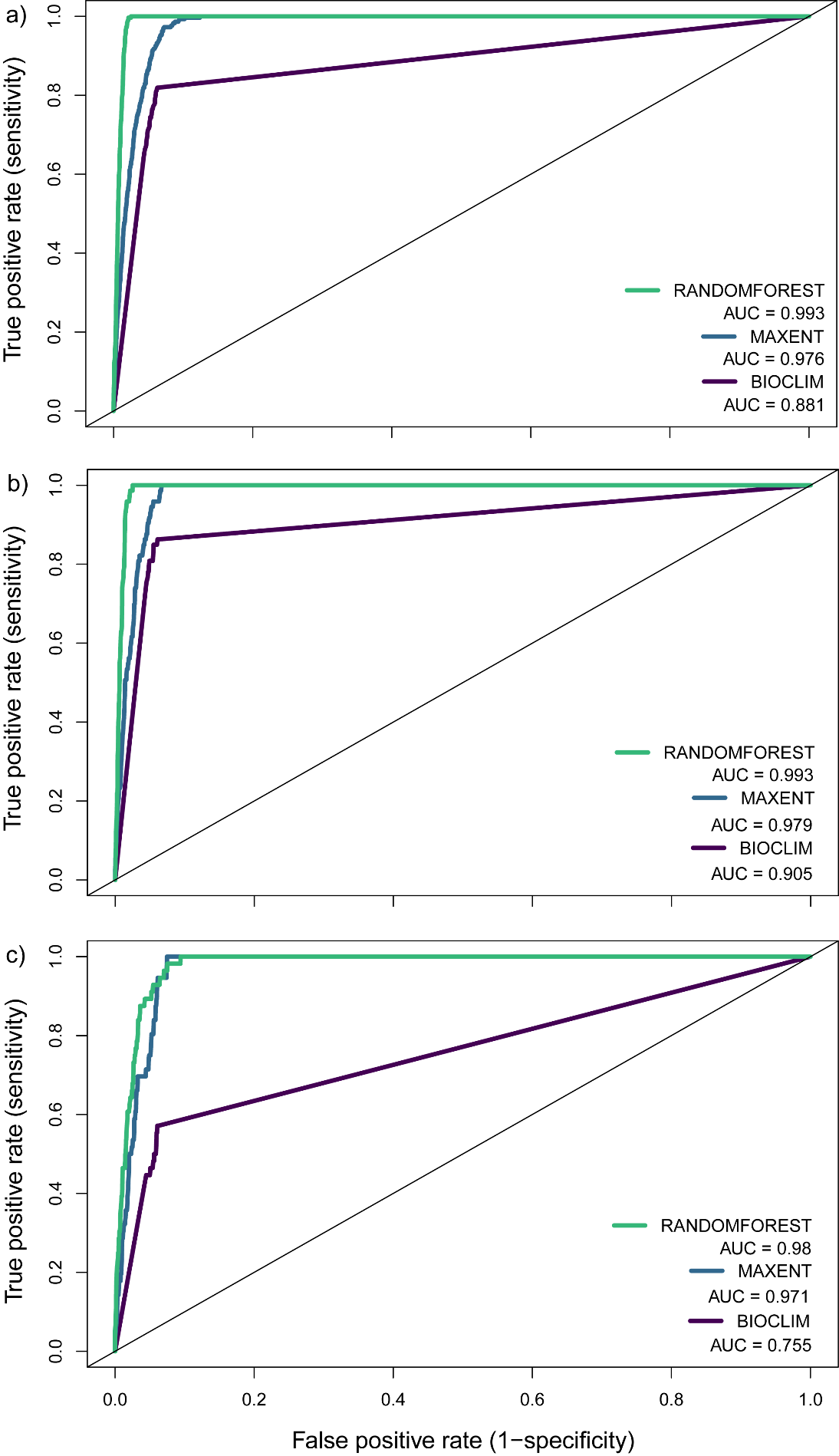


Figure SM 12. Receiver operating characteristic (ROC) curves from mean model predictions for each model algorithm, trained on the modern occurrence dataset. (a) ROC curves and AUC values generated using the modern occurrence dataset and modern projection. (b) ROC curves and AUC values generated using the modern test dataset and modern projection. (c) ROC curves and AUC values generated using LIG occurrences as an evaluation dataset with the LIG hindcasting projection.


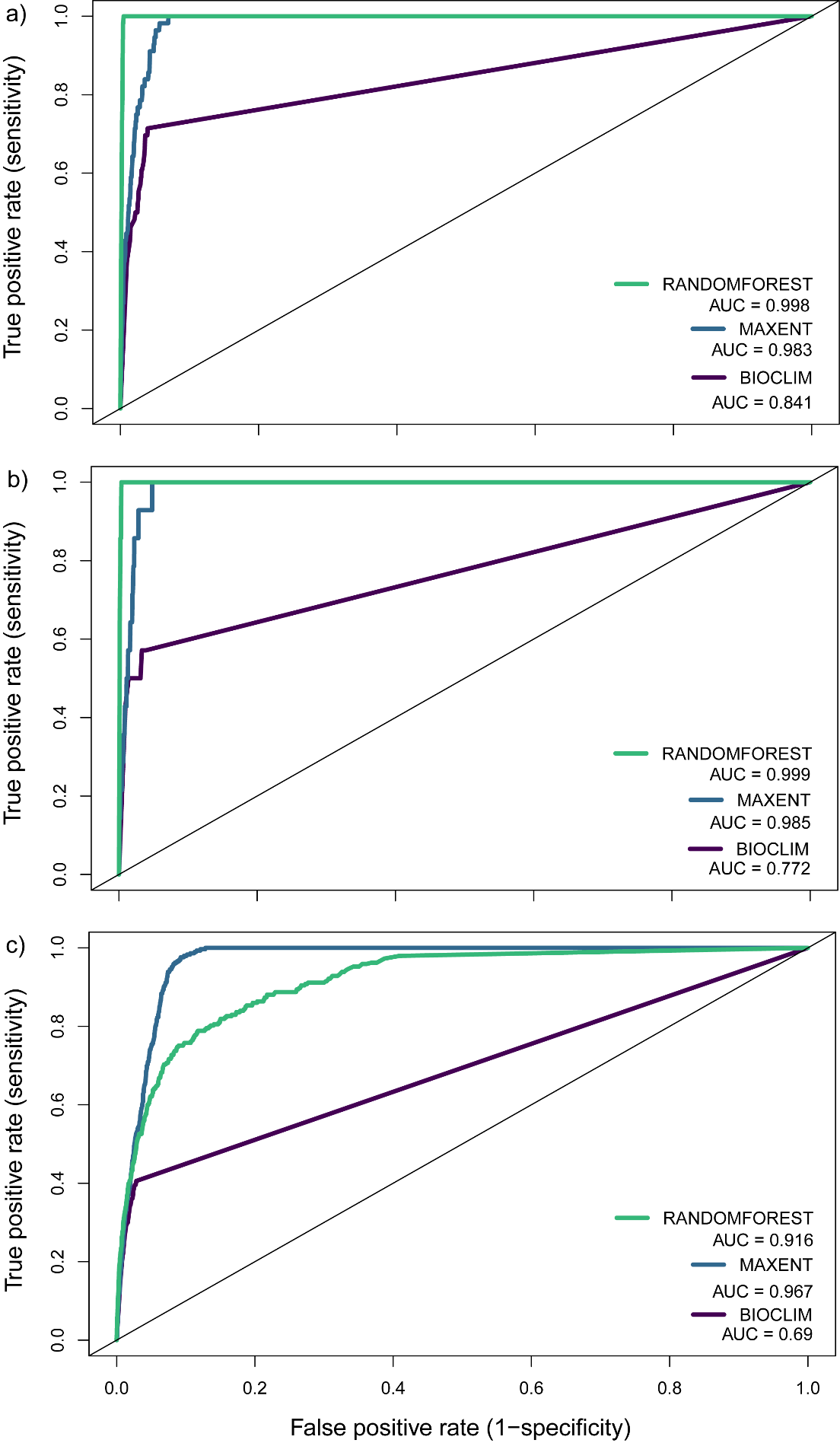


Figure SM 13. Receiver operating characteristic (ROC) curves from mean model predictions for each model algorithm, trained on the LIG occurrence dataset. (a) ROC curves and AUC values generated using the LIG occurrence dataset and LIG projection. (b) ROC curves and AUC values generated using the LIG test dataset and LIG projection. (c) ROC curves and AUC values generated using LIG occurrences as an evaluation dataset with the modern forecasting projection.

**
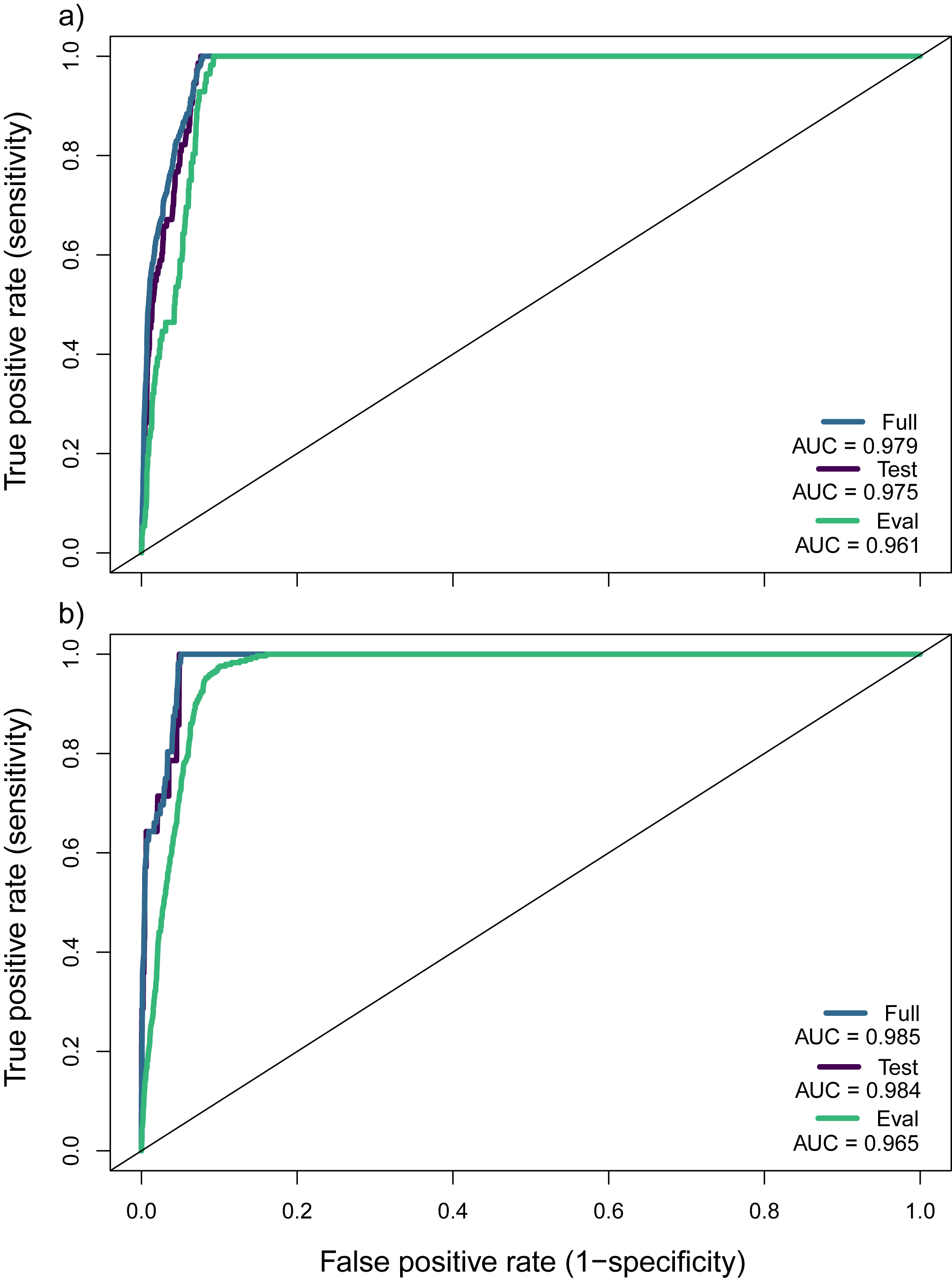
**

Figure SM 14. Receiver operating characteristic (ROC) curves for the mean predictions made from all model algorithms from both training datasets. (a) ROC curves and AUC values for model predictions trained on the modern occurrence dataset. The different ROC curves show values associated with test occurrence dataset, full occurrence dataset and evaluation dataset (LIG occurrence and LIG projection). (b) ROC curves and AUC values for model predictions trained on the LIG occurrence dataset. The different ROC curves show values associated with test occurrence dataset, full occurrence dataset and evaluation dataset (modern occurrence and modern projection).

## Variable contribution

To provide insight into the importance of each environmental variable for the development of our ENMs, we employed biomod2’s variable importance test. Based on the results of this test, bathymetry is consistently the most important variable for all algorithms and training datasets for model fitting, followed by annual sea surface temperature (Table SM 3). Annual salinity and irradiance variables seem relatively unimportant for model fitting using the MaxEnt algorithm, whilst having particularly more importance for the BIOCLIM algorithm for both training datasets.

Table SM 3. Mean biomod2 variable importance test scores for ecological niche models trained on both modern and LIG training datasets, for all modelling algorithms implemented. These results display 1 - cor (Pearson correlation coefficient score) between predictions based on calibrated models, and predictions after one environmental variable is randomised. Higher values indicate higher importance in model calibration, whist lower values indicate less importance.

| **MODERN** | **ENVIRONMENTAL VARIABLE** | **MODELLING ALGORITHM** | | |
| --- | --- | --- | --- | --- |
|  |  | **MAXENT** | **BIOCLIM** | **RANDOMFOREST** |
|  | Bathymetry | 0.86229 | 0.83352 | 0.79289 |
|  | December, January, February irradiance | 0.01895 | 0.16982 | 0.08015 |
|  | June, July, August irradiance | 0.08996 | 0.29486 | 0.09462 |
|  | Annual salinity | 0.02147 | 0.1025 | 0.06003 |
|  | Annual sea surface temperature | 0.53401 | 0.38374 | 0.44529 |
|  |  |  | | |
|  |  |  | | |
| **LIG** | **ENVIRONMENTAL VARIABLE** | **MODELLING ALGORITHM** | | |
|  |  | **MAXENT** | **BIOCLIM** | **RANDOMFOREST** |
|  | Bathymetry | 0.84902 | 0.87536 | 0.67951 |
|  | December, January, February irradiance | 0.03225 | 0.17303 | 0.22486 |
|  | June, July, August irradiance | 0.06195 | 0.37765 | 0.20744 |
|  | Annual salinity | 0.04829 | 0.23778 | 0.08172 |
|  | Annual sea surface temperature | 0.56119 | 0.41206 | 0.47094 |

## Geographic predictions & clamping masks

Below, we provide the raw outputs for each model prediction covering all predictions (Figure SM 15–16). These figures include all results from each algorithm, projection and training dataset. The raw outputs represent the unclamped model predictions and provide no account of uncertainties. From these figures it is clear to see the variation in assigned habitability scores, with 0 indicating the lowest habitability and 1 the highest. In addition, we also provide the binary predictions for each threshold (5LPT, 10LPT, 20LPT) and climate scenario (Figure SM17–20). Clamping masks for each model prediction are also provided (Figure SM 21–22). These masks identify locations where predictions are uncertain as the values of environmental variables are outside the range used for model calibration. The values indicated by these masks relates to the number of variables outside the calibration range.


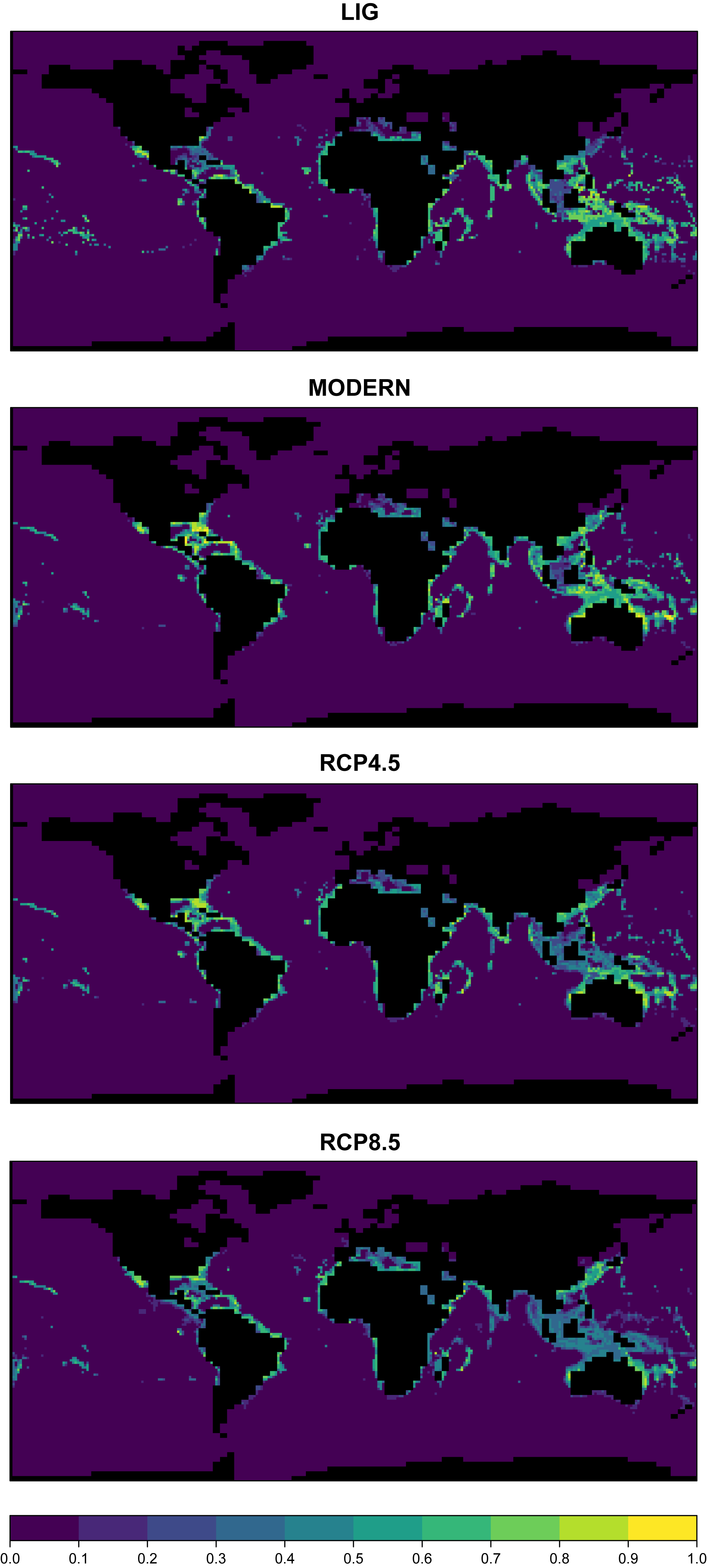


Figure SM 15. Global mean raw habitability map outputs (unclamped) for all climate scenarios, trained on modern occurrences.


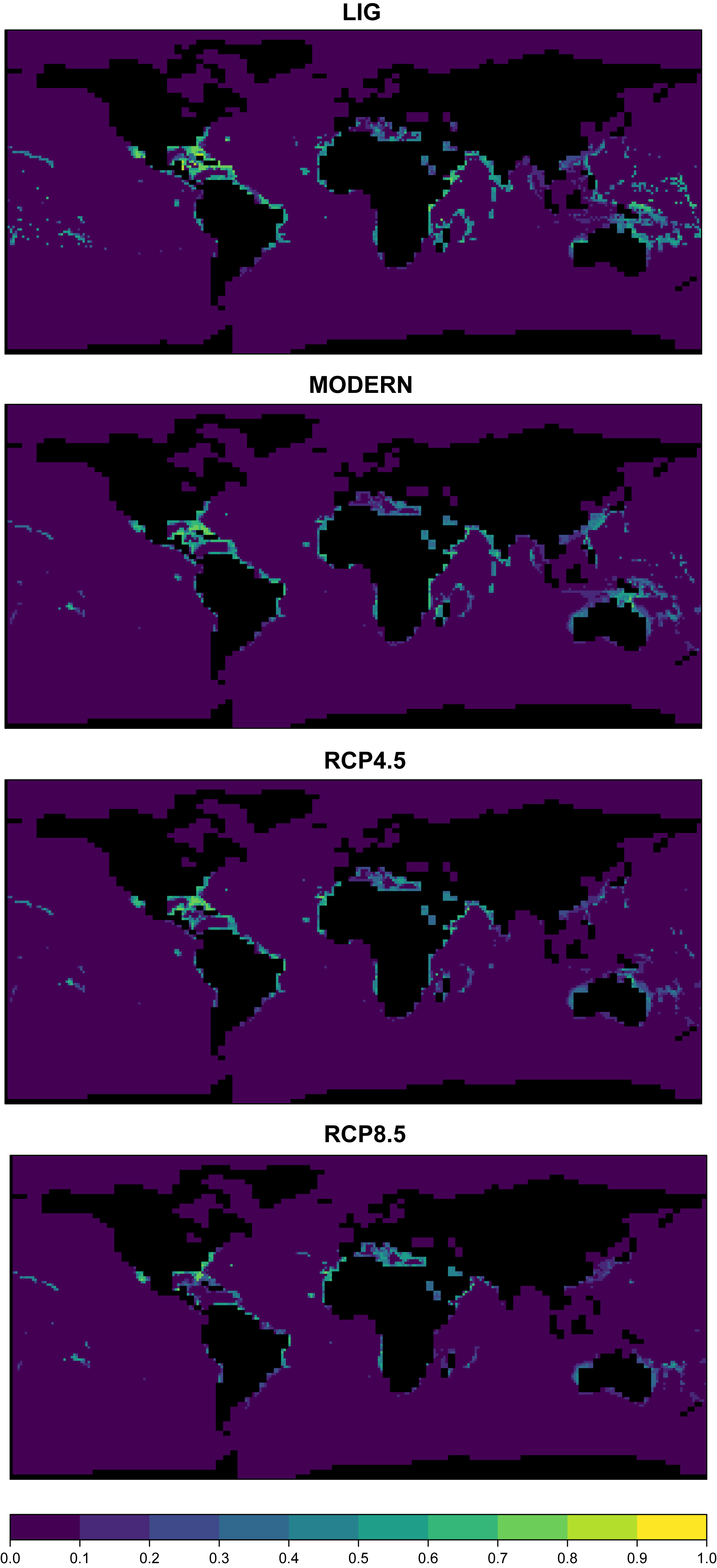


Figure SM 16. Global mean raw habitability map outputs (unclamped) for all climate scenarios, trained on LIG occurrences.

*
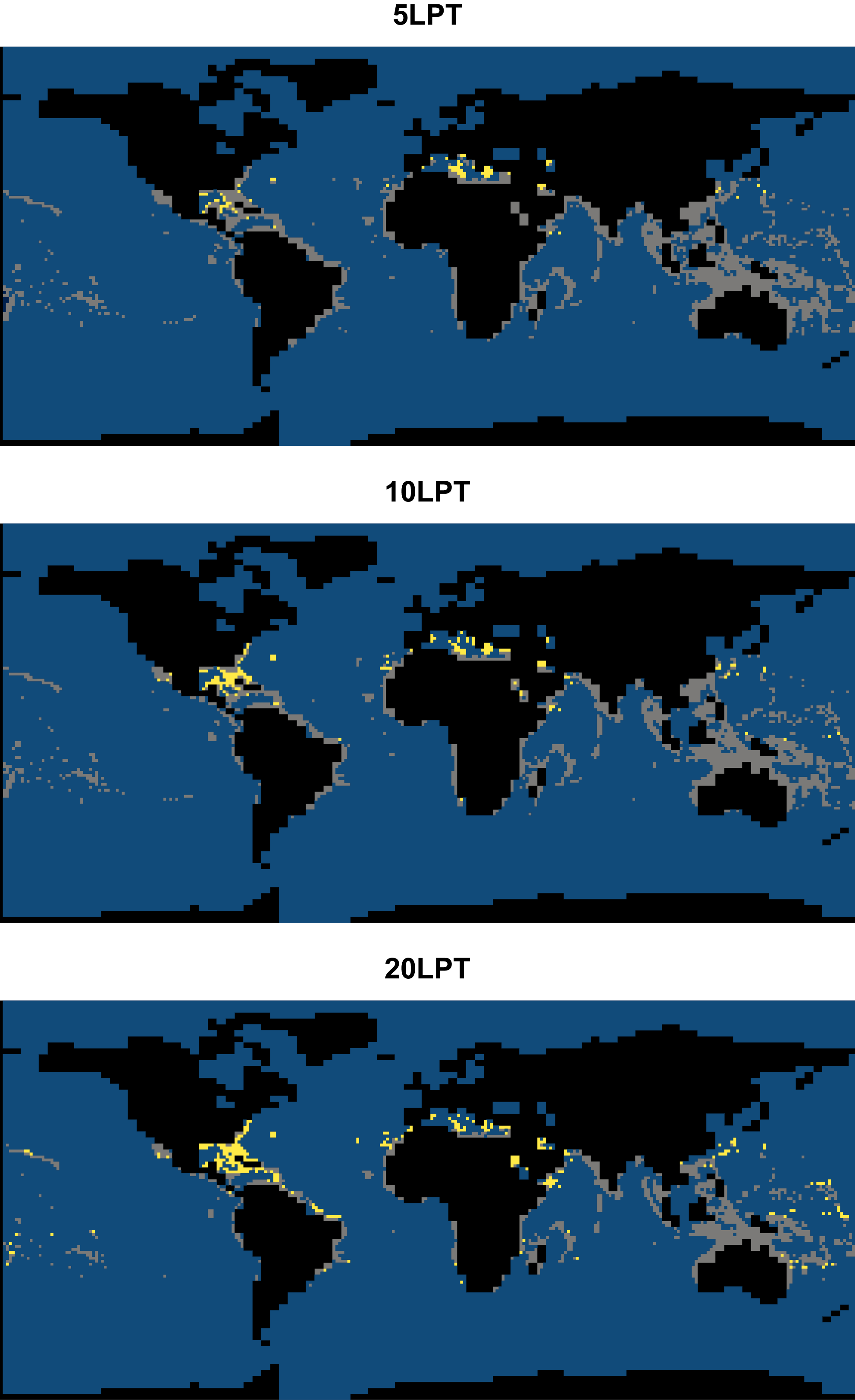
*

Figure SM 17. Combined binary habitability maps for the LIG using the three binary thresholds (5LPT, 10LPT, and 20LPT). The blue indicates unsuitable cells, whilst grey and yellow indicate suitable habitats. Specifically, grey illustrates habitable cells predicted by the modern occurrence trained models, whilst yellow highlights additional cells predicted for those trained on the LIG occurrence data.


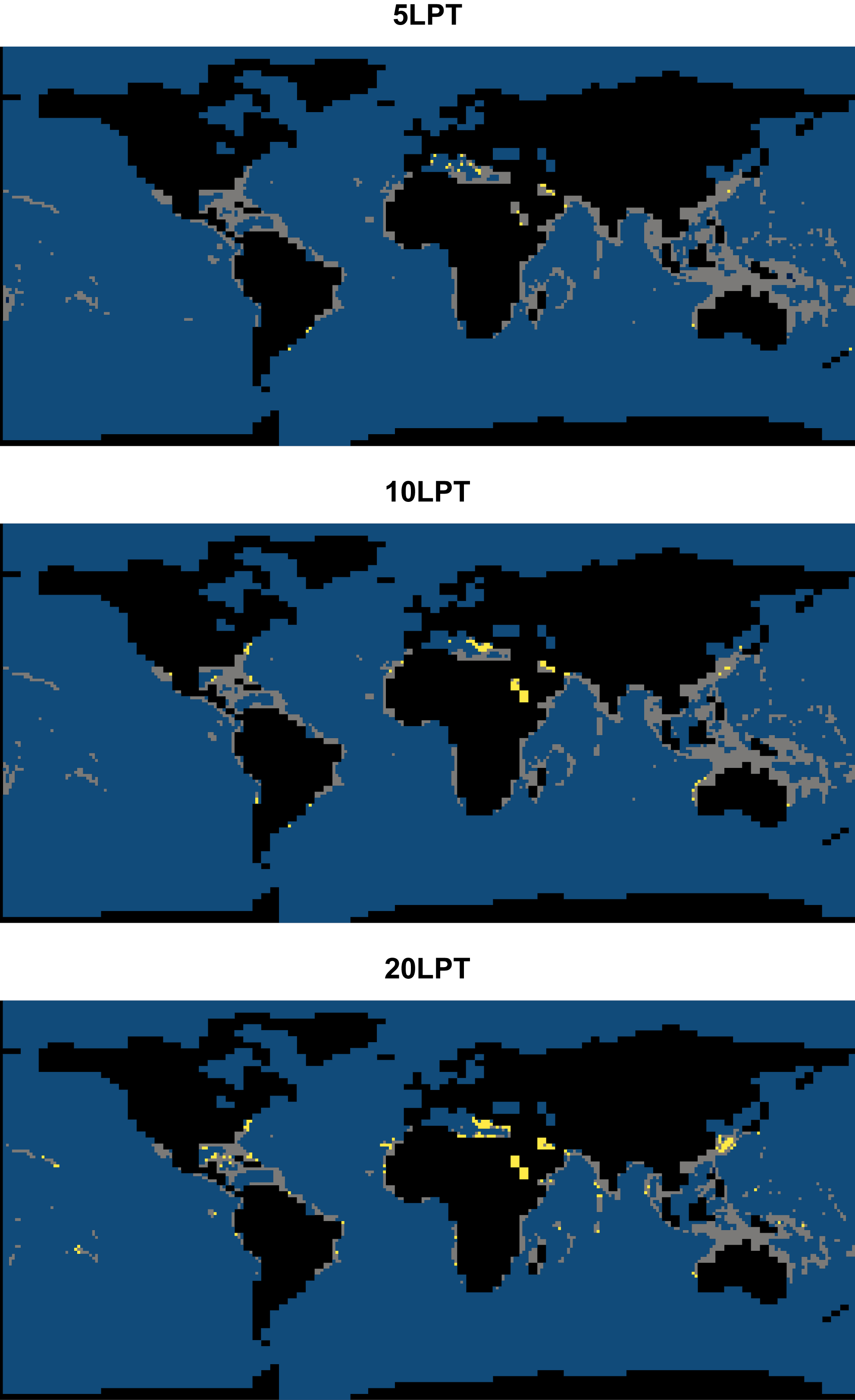


Figure SM 18. Combined binary habitability maps for the modern using the three binary thresholds (5LPT, 10LPT, and 20LPT). The blue indicates unsuitable cells, whilst grey and yellow indicate suitable habitats. Specifically, grey illustrates habitable cells predicted by the modern occurrence trained models, whilst yellow highlights additional cells predicted for those trained on the LIG occurrence data.


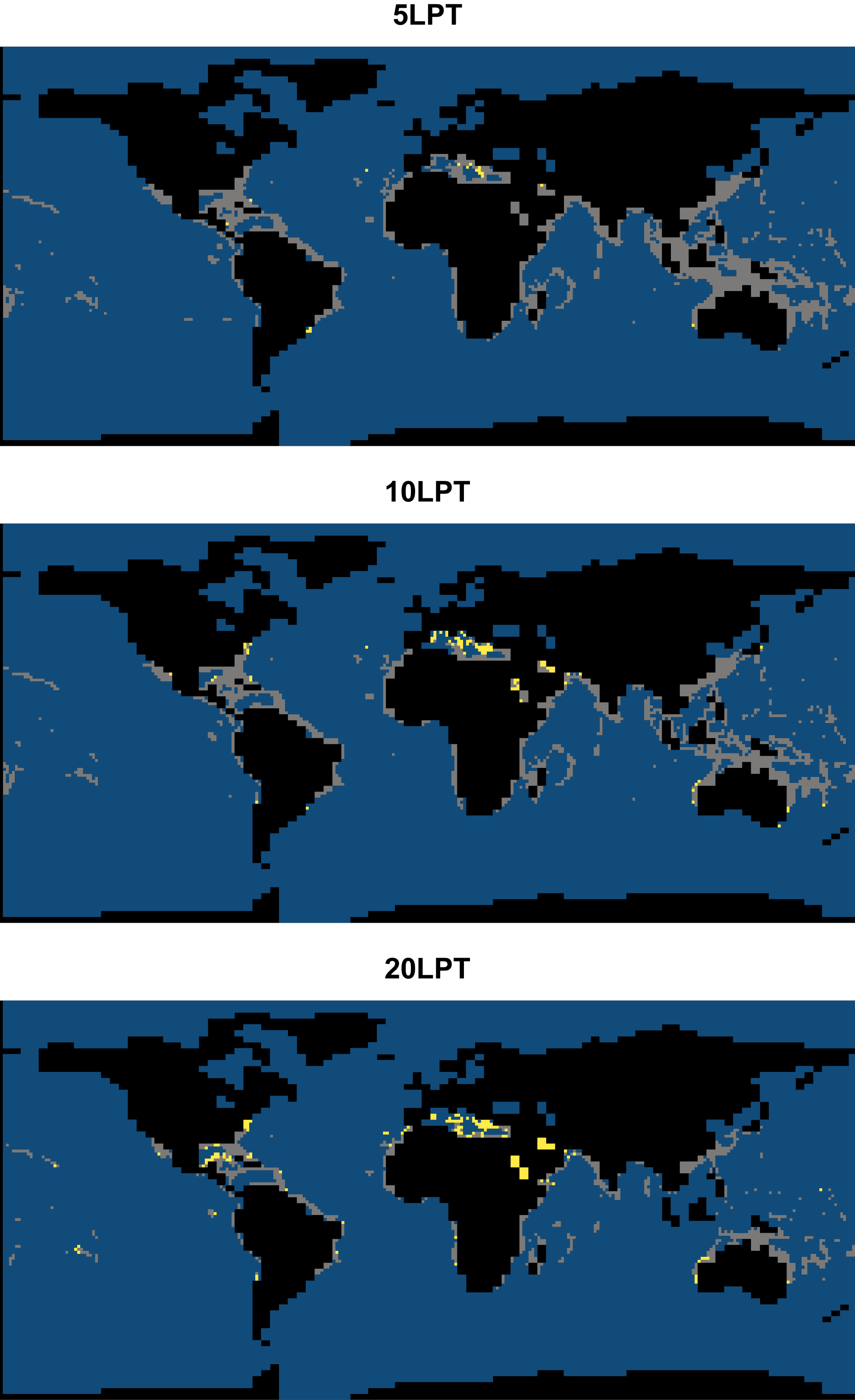


Figure SM 19. Combined binary habitability maps for RCP4.5 using the three binary thresholds (5LPT, 10LPT, and 20LPT). The blue indicates unsuitable cells, whilst grey and yellow indicate suitable habitats. Specifically, grey illustrates habitable cells predicted by the modern occurrence trained models, whilst yellow highlights additional cells predicted for those trained on the LIG occurrence data.


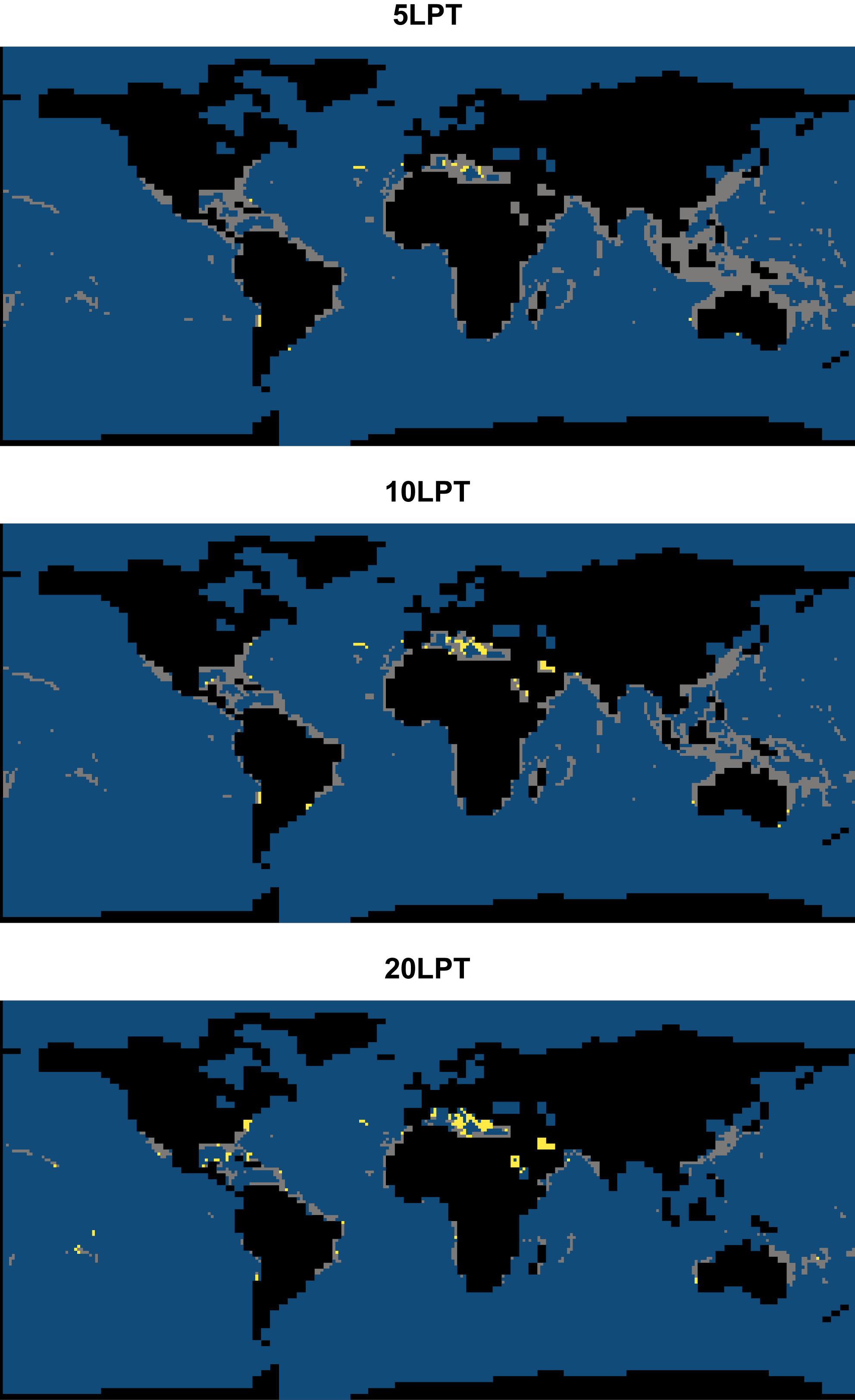


Figure SM 20. Combined binary habitability maps for RCP8.5 using the three binary thresholds (5LPT, 10LPT, and 20LPT). The blue indicates unsuitable cells, whilst grey and yellow indicate suitable habitats. Specifically, grey illustrates habitable cells predicted by the modern occurrence trained models, whilst yellow highlights additional cells predicted for those trained on the LIG occurrence data.

**
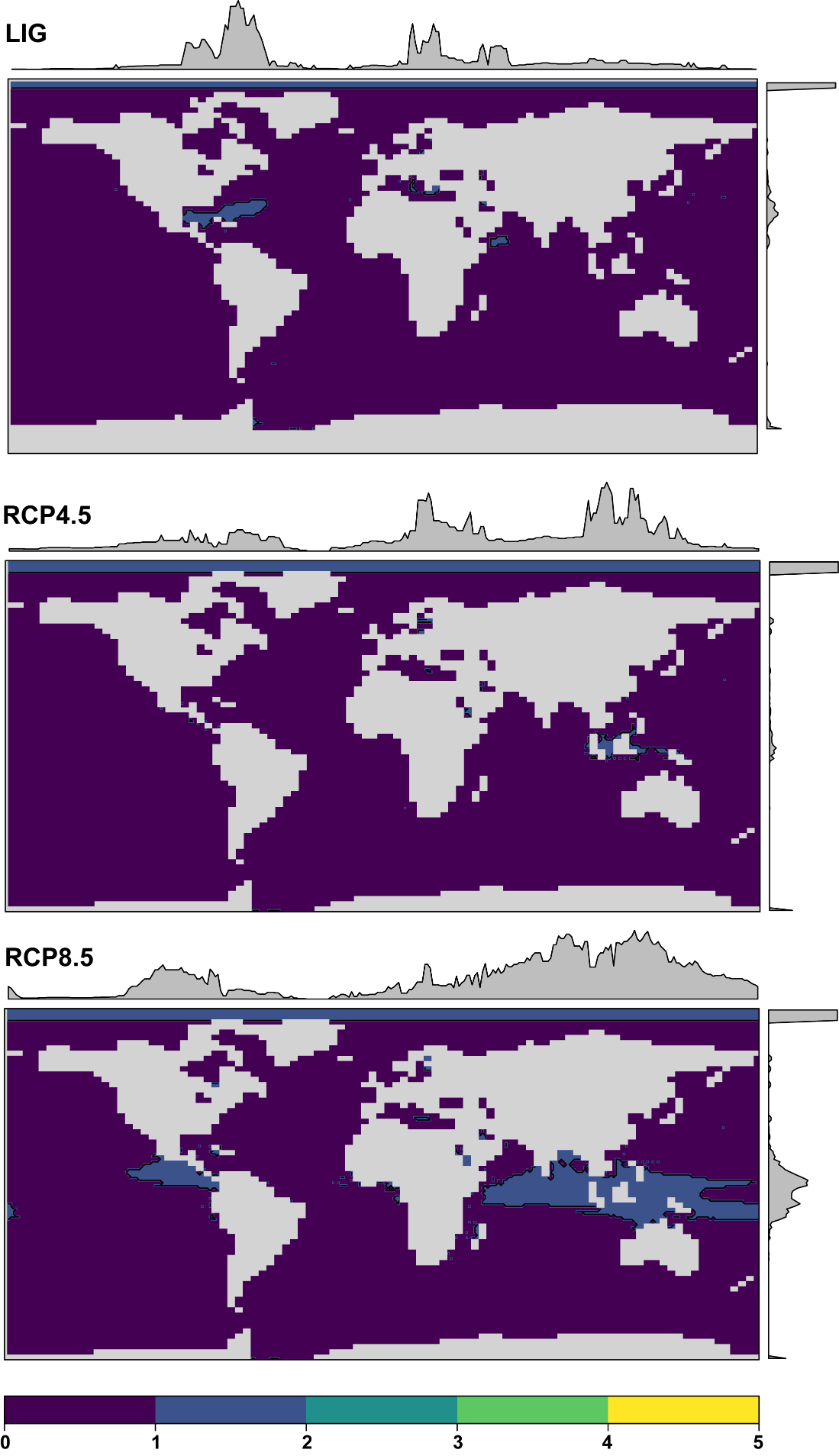
**

Figure SM 21. Clamping masks for modern trained predictions. These masks identify locations where predictions are uncertain as the values of environmental variables are outside the range used for model calibration. The values indicated by these masks relates to the number of variables outside the calibration range.

**
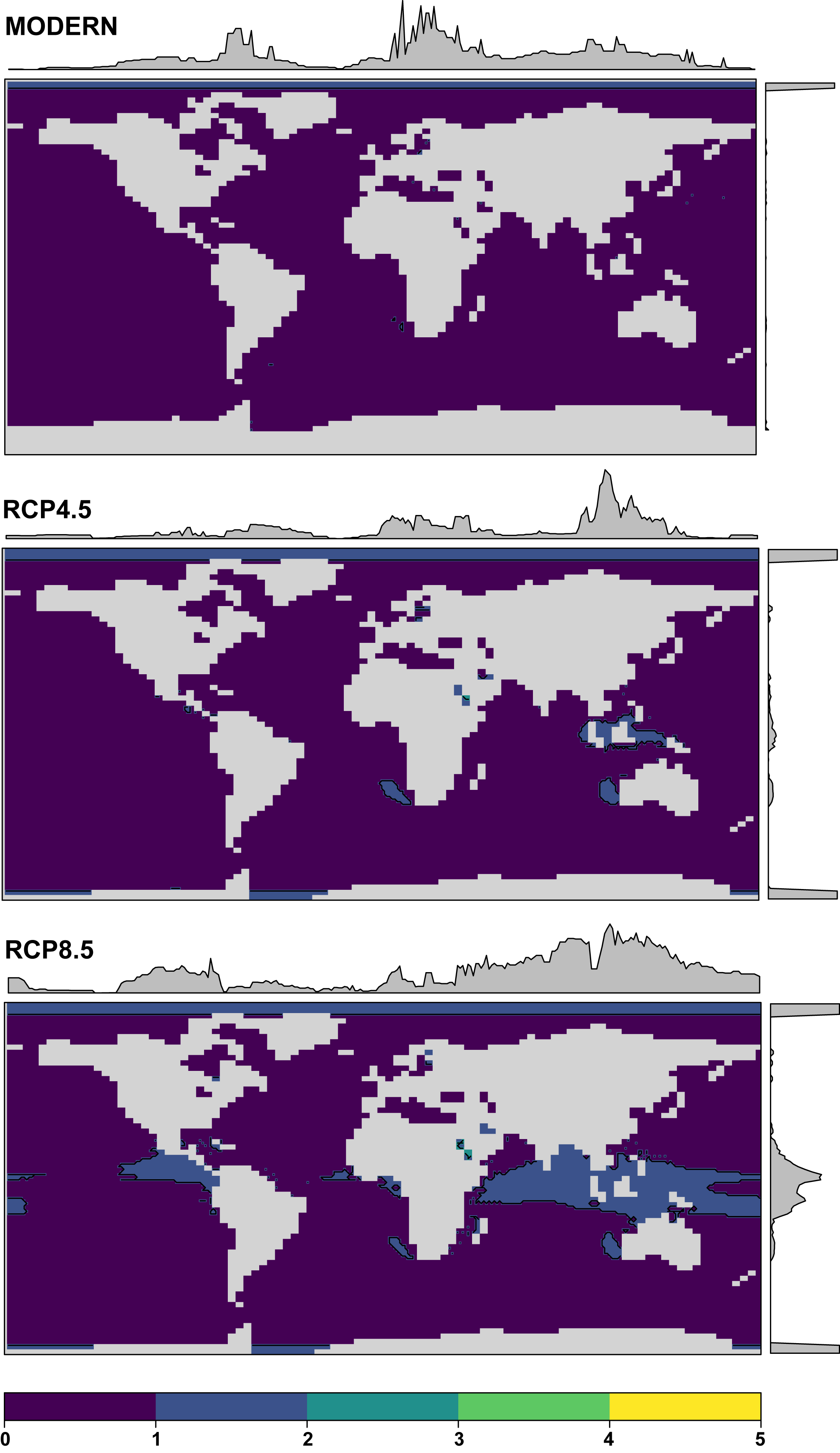
**

Figure SM 22. Clamping masks for LIG trained predictions. These masks identify locations where predictions are uncertain as the values of environmental variables are outside the range used for model calibration. The values indicated by these masks relates to the number of variables outside the calibration range.

**
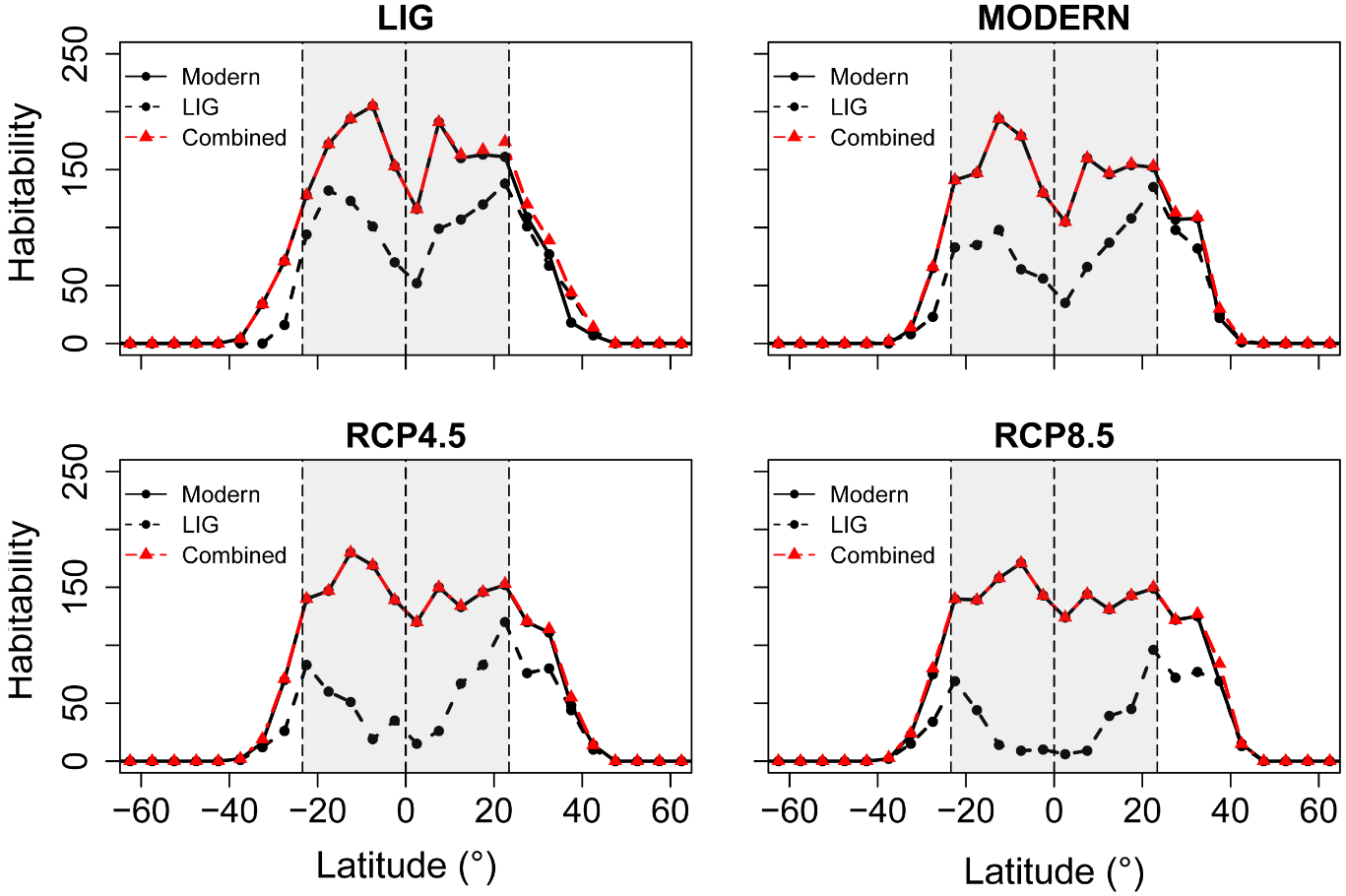
**

Figure SM 23. Binned latitudinal habitability for 5LPT. In this instance, habitability is the sum of cells designated as habitable by all three model classes within 5° latitudinal bins. Solid black line indicates output trained on the modern occurrence dataset, whilst the dashed line indicates output trained on the LIG occurrence dataset. The dashed red line represents the combined outputs. Grey shaded area represents the extent of the tropics in the present day. Dashed lines indicate the equator, the Northern Tropic, and the Southern Tropic.


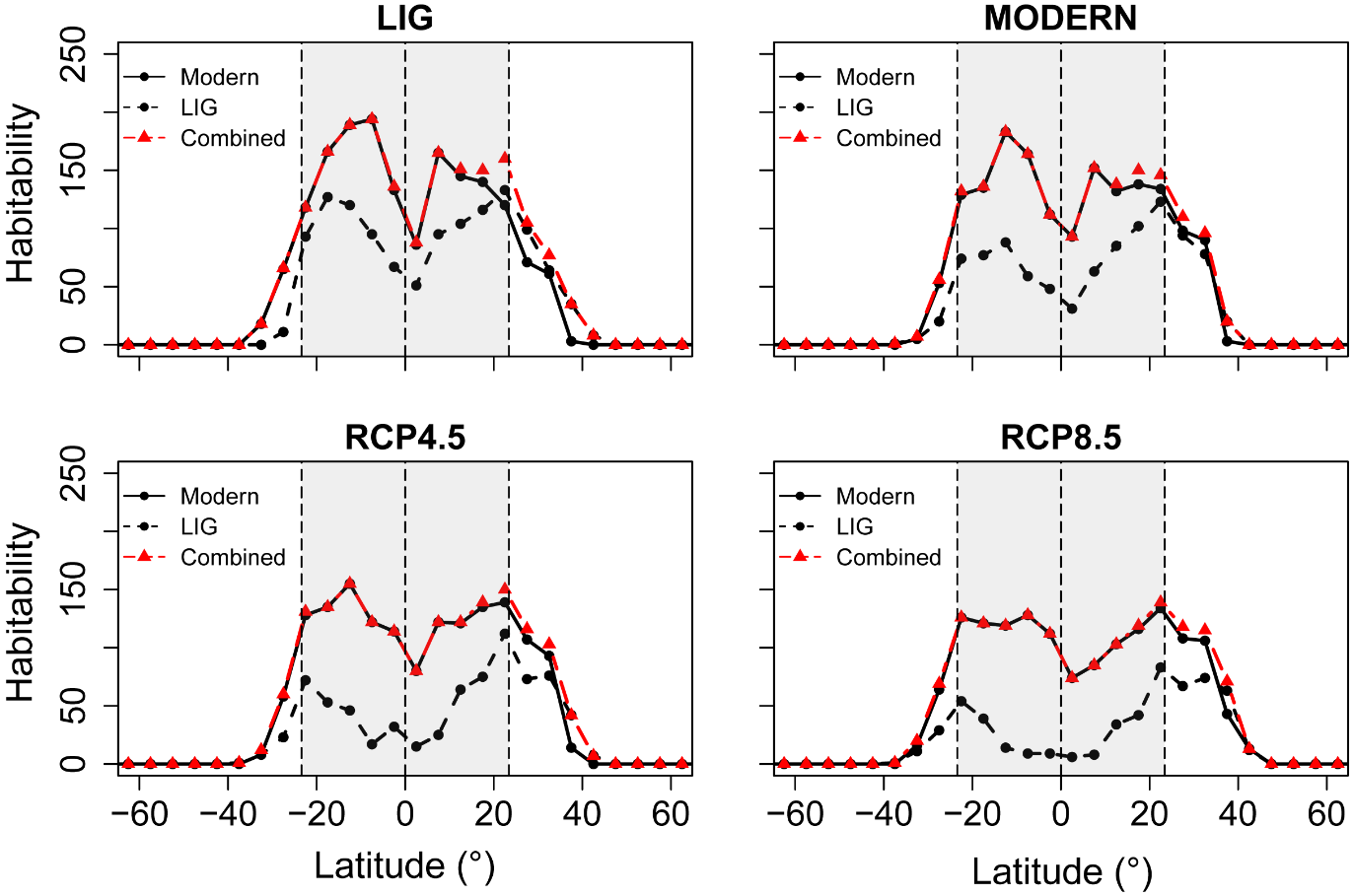


Figure SM 24. Binned latitudinal habitability for 10LPT. In this instance, habitability is the sum of cells designated as habitable by all three model classes within 5° latitudinal bins. Solid black line indicates output trained on the modern occurrence dataset, whilst the dashed line indicates output trained on the LIG occurrence dataset. The dashed red line represents the combined outputs. Grey shaded area represents the extent of the tropics in the present day. Dashed lines indicate the equator, the Northern Tropic, and the Southern Tropic.


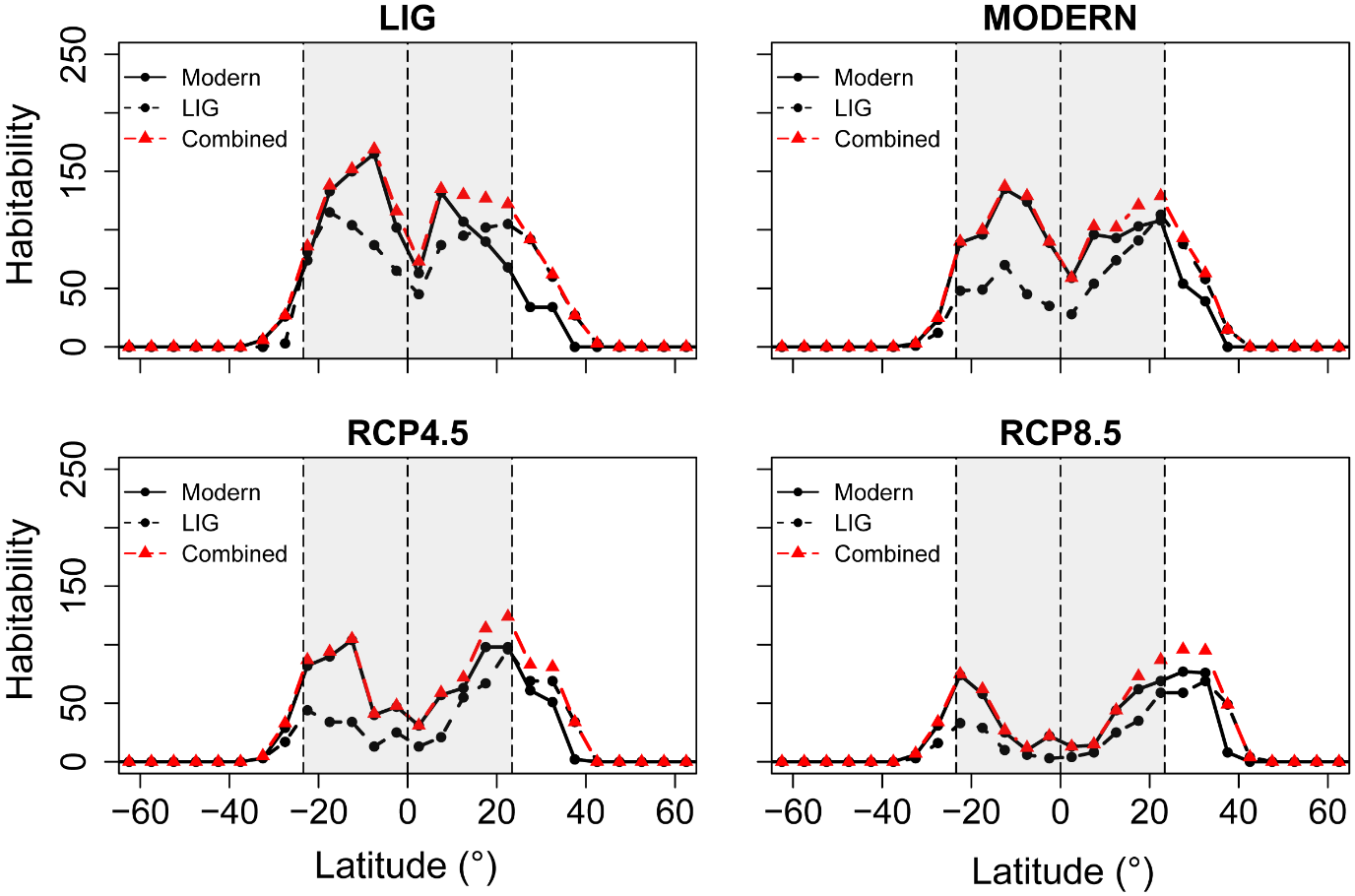


Figure SM 25. Binned latitudinal habitability for 20LPT. In this instance, habitability is the sum of cells designated as habitable by all three model classes within 5° latitudinal bins. Solid black line indicates output trained on the modern occurrence dataset, whilst the dashed line indicates output trained on the LIG occurrence dataset. The dashed red line represents the combined outputs. Grey shaded area represents the extent of the tropics in the present day. Dashed lines indicate the equator, the Northern Tropic, and the Southern Tropic.

# References

[1] Flato, G., Marotzke, J., Abiodun, B., Braconnot, P., Chou, S., Collins, W., Cox, P.M., Driouech, F., Emori, S., Eyring, V., et al. 2013 Evaluation of Climate Models. In *Climate Change 2013: The Physical Science Basis. Contribution of Working Group I to the Fifth Assessment Report of the Intergovernmental Panel on Climate Change* (eds. T. Qin, D. Plattner, M. Tignor, S. Allen, J. Boschung, A. Nauels, Y. Xia, V. Bex & P. Midgley), pp. 741-866. Cambridge, UK, Cambridge University Press.

[2] Wiens, J.A., Stralberg, D., Jongsomjit, D., Howell, C.A. & Snyder, M.A. 2009 Niches, models, and climate change: assessing the assumptions and uncertainties. *Proc Natl Acad Sci U S A* **106 Suppl 2**, 19729-19736. (doi:10.1073/pnas.0901639106).

[3] Couce, E., Ridgwell, A. & Hendy, E.J. 2012 Environmental controls on the global distribution of shallow-water coral reefs. *Journal of Biogeography* **39**, 1508-1523. (doi:10.1111/j.1365-2699.2012.02706.x).

[4] van Vuuren, D.P., Edmonds, J., Kainuma, M., Riahi, K., Thomson, A., Hibbard, K., Hurtt, G.C., Kram, T., Krey, V., Lamarque, J.-F., et al. 2011 The representative concentration pathways: an overview. *Climatic Change* **109**, 5-31. (doi:10.1007/s10584-011-0148-z).

[5] Riahi, K., Rao, S., Krey, V., Cho, C., Chirkov, V., Fischer, G., Kindermann, G., Nakicenovic, N. & Rafaj, P. 2011 RCP 8.5—A scenario of comparatively high greenhouse gas emissions. *Climatic Change* **109**, 33-57. (doi:10.1007/s10584-011-0149-y).

[6] Thomson, A.M., Calvin, K.V., Smith, S.J., Kyle, G.P., Volke, A., Patel, P., Delgado-Arias, S., Bond-Lamberty, B., Wise, M.A., Clarke, L.E., et al. 2011 RCP4.5: a pathway for stabilization of radiative forcing by 2100. *Climatic Change* **109**, 77-94. (doi:10.1007/s10584-011-0151-4).

[7] Clark, P.U. & Huybers, P. 2009 Interglacial and future sea level. *Nature* **462**, 856-857. (doi:10.1029/2009gl040222).

[8] McKay, N.P., Overpeck, J.T. & Otto-Bliesner, B.L. 2011 The role of ocean thermal expansion in Last Interglacial sea level rise. *Geophysical Research Letters* **38**, n/a-n/a. (doi:10.1029/2011gl048280).

[9] Dutton, A. & Lambeck, K. 2012 Ice Volume and Sea Level During the Last Interglacial. *Science* **337**, 216-219.

[10] Stirling, C.H., Esat, T., Lambeck, K. & McCulloch, M.T. 1998 Timing and duration of the Last Interglacial: evidence for a restricted interval of widespread coral reef growth. *Earth and Planetary Science Letters* **160**, 745-762.

[11] Veeh, H.H. 1966 Th^230^/U^238^ and U^234^/U^238^ ages of Pleistocene high sea level stand. *Journal of Geophysical Research* **71**, 3379-3386.

[12] Jiang, L.Q. & Feely, R.A. 2015 Aragonite saturation state gridded to 1x1 degree latitude and longitude at depth levels of 0, 50, 100, 200, 500, 1000, 2000, 3000, and 4000 meters in the global oceans (NCEI Accession 0139360). Version 1.1. (NOAA National Centers for Environmental Information. .
